# FELLA: an R package to enrich metabolomics data

**DOI:** 10.1186/s12859-018-2487-5

**Published:** 2018-12-22

**Authors:** Sergio Picart-Armada, Francesc Fernández-Albert, Maria Vinaixa, Oscar Yanes, Alexandre Perera-Lluna

**Affiliations:** 1 B2SLab, Departament d’Enginyeria de Sistemes, Automàtica i Informàtica Industrial, Universitat Politècnica de Catalunya, Barcelona, 08028 Spain; 2 Networking Biomedical Research Centre in the subject area of Bioengineering, Biomaterials and Nanomedicine (CIBER-BBN), Madrid, 28029 Spain; 3 Institut de Recerca Pediàtrica Hospital Sant Joan de Déu, Esplugues de Llobregat, Barcelona, 08950 Spain; 4 Metabolomics Platform, IISPV, Department of Electronic Engineering (DEEEA), Universitat Rovira i Virgili, Tarragona, 43003 Spain; 5 CIBER of Diabetes and Associated Metabolic Diseases (CIBERDEM), Madrid, 28029 Spain; 6 Takeda Cambridge Ltd, Cambridge, CB4 0PZ UK

**Keywords:** Metabolomics, Pathways, Network analysis, Data mining, Knowledge representation

## Abstract

**Background:**

Pathway enrichment techniques are useful for understanding experimental metabolomics data. Their purpose is to give context to the affected metabolites in terms of the prior knowledge contained in metabolic pathways. However, the interpretation of a prioritized pathway list is still challenging, as pathways show overlap and cross talk effects.

**Results:**

We introduce FELLA, an R package to perform a network-based enrichment of a list of affected metabolites. FELLA builds a hierarchical representation of an organism biochemistry from the Kyoto Encyclopedia of Genes and Genomes (KEGG), containing pathways, modules, enzymes, reactions and metabolites. In addition to providing a list of pathways, FELLA reports intermediate entities (modules, enzymes, reactions) that link the input metabolites to them. This sheds light on pathway cross talk and potential enzymes or metabolites as targets for the condition under study. FELLA has been applied to six public datasets –three from *Homo sapiens*, two from *Danio rerio* and one from *Mus musculus*– and has reproduced findings from the original studies and from independent literature.

**Conclusions:**

The R package FELLA offers an innovative enrichment concept starting from a list of metabolites, based on a knowledge graph representation of the KEGG database that focuses on interpretability. Besides reporting a list of pathways, FELLA suggests intermediate entities that are of interest per se. Its usefulness has been shown at several molecular levels on six public datasets, including human and animal models. The user can run the enrichment analysis through a simple interactive graphical interface or programmatically. FELLA is publicly available in Bioconductor under the GPL-3 license.

**Electronic supplementary material:**

The online version of this article (10.1186/s12859-018-2487-5) contains supplementary material, which is available to authorized users.

## Background

Metabolomics is the science that measures lightweight molecules in living organisms and stands as a valuable source of biomarkers and biological knowledge [1]. The preprocessing of such data can be achieved through pipelines like MeltDB [2] or MAIT [3]. Once metabolite abundances are available, pathway analysis tools ease data interpretation [4] by framing the affected metabolites in terms of contextual knowledge. Databases like the Kyoto Encyclopedia of Genes and Genomes (KEGG) [5] are sources of curated pathway data. The classification of enrichment techniques used here follows the review in [4].

Over representation analysis (ORA) approaches are based on testing the proportion of a list of affected metabolites inside a pathway. ORA is available in tools like the web server MetaboAnalyst [6] and the R package *clusterProfiler* [7]. Functional class scoring (FCS) approaches use quantitative data instead and seek subtle but coordinated changes in the metabolites belonging to a pathway. MSEA in MetaboAnalyst and IMPaLA [8] contain implementations of FCS for metabolomics. Pathway topology-based (PT) approaches further include topological measures of the metabolites in the statistic, accounting for their inequivalence within the pathway. PT analyses can be performed using MetaboAnalyst.

Here, we introduce the R package *FELLA*, available in Bioconductor [9], for metabolomics data interpretation that combines pathway enrichment with network analysis. The list of affected metabolites and the reported pathways are connected through intermediate entities -reactions, enzymes, modules- in a heterogeneous network layout. This suggests how the perturbation spreads at the pathway level and how pathways cross talk, enhancing the interpretability of the output.

## Implementation

**Fig. 1:**
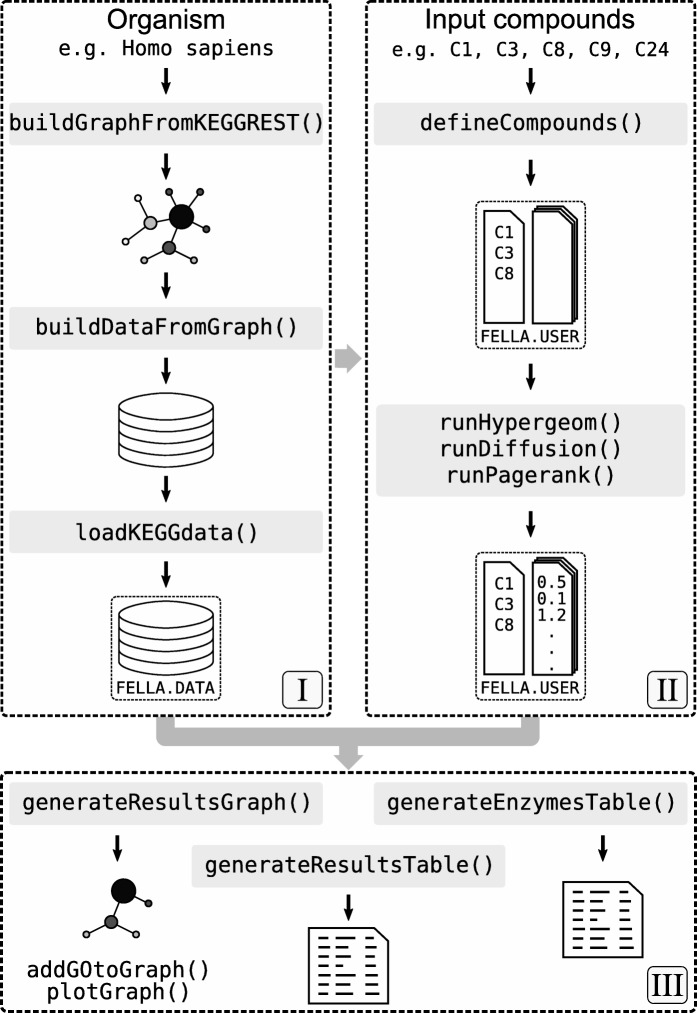
Design of the R package *FELLA*. (I) creation of a graph object from an organism code and its database, (II) ID mapping and propagation algorithms (diffusion, PageRank) to score all the nodes, (III) node prioritisation and results exporting

*FELLA* is an R package that performs metabolomics data enrichment starting from (I) a network derived from KEGG and (II) a list of KEGG compounds (Fig. 1). A sub-network relevant to the input is extracted from (I) using network propagation algorithms that start from the labels in (II), providing a data enrichment that goes beyond a pathway list. The purpose of *FELLA* is to elaborate a biological explanation that justifies how the input metabolites can reach the reported pathways, as well as perspective on pathway cross talk. Two user guides illustrate the principles and the usage of *FELLA*: a quickstart (Additional file 1) and an in-depth vignette with implementations details and three real examples (Additional file 2). Two additional vignettes (Additional files 3 and 4) serve as case studies for non-human organisms.

### Methodology

**Fig. 2:**
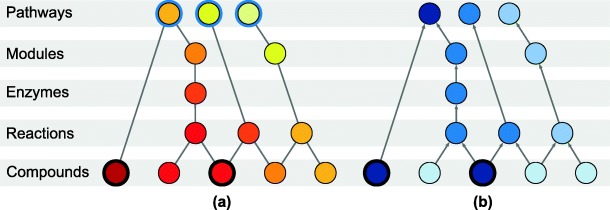
Node arrangement for the knowledge model used by *FELLA*. Entities are organised in a hierarchical manner, from bottom to top: KEGG compounds or metabolites, reactions, enzymes, KEGG modules and pathways. Binary labels at the level of metabolites are propagated to the rest of the network and a relevant, small sub-network is automatically reported. Nodes are ranked using the network propagation algorithms **a** heat diffusion and **b** PageRank. The affected metabolites are highlighted with a black ring. For heat diffusion (**a**), affected metabolites are forced to generate unitary flow. Every pathway is highlighted with a blue ring, representing its connection to a cool boundary node. In equilibrium, the highest temperature pathways (and nodes) will have the greatest heat flow, suggesting a relevant role in the experiment. For PageRank (**b**), affected metabolites are the start of random walks. PageRank scores, represented by the intensity of the blue colour, will attain higher values in the frequently reached random walk nodes. Figure extracted from [12]

The cornerstone of *FELLA* is its knowledge graph representation of the biochemistry in KEGG at several molecular levels. The network is hierarchical and connects KEGG compounds (metabolites) to KEGG pathways through intermediate entities, namely reactions, enzymes and KEGG modules, see Fig. 2. Such connections (edges) are obtained directly from KEGG annotations. The presence of intermediate levels allows inference at their level, meaning that relevant reactions, enzymes and KEGG modules can be suggested just by starting from a list of affected metabolites. This feature is evaluated in several case studies, by linking the suggested enzymatic families and reactions to literature and to original findings within the studies.

In order to report a sub-network, nodes are ranked according to a scoring function –based on network propagation– and only the top scoring nodes are returned. Two algorithms are supported for propagating the labels from the affected metabolites: a classical heat diffusion approach [10] and the PageRank web ranking algorithm [11]. Further details on the network propagation settings can be found in [12] and in Additional file 2. The main difference between both algorithms is that heat diffusion is undirected whereas PageRank is directed upwards. In practice, contrary to PageRank, heat diffusion will frequently report new metabolites because heat is allowed to propagate back to compounds from the upper levels [12]. This behaviour can ease the discovery of intermediate metabolites that lay close to the input metabolites and tend to connect them. An example of its usefulness can be found in the gilt-head bream study.

As exposed in [12], ranking nodes according to their raw diffusion scores suffers from a strong bias, related to the node level and topological features. This is addressed by normalising the diffusion score of every node using its background distribution under input permutations. Permutations can be simulated through Monte Carlo trials to obtain an empirical p-value, labelled as p-score. Alternatively, a parametric z-score can be obtained without requiring Monte Carlo trials. The p-score is obtained by transforming the z-score to lie in the [0,1] interval through the cumulative distribution function of a standard normal distribution. Under both statistical approximations, nodes with the lowest p-scores are reported as the suggested sub-network. Note that p-scores are used as a ranker rather than for testing hypotheses.

An optional filter allows the removal of small connected components from the reported sub-network. When building the database, a number of random sub-networks are sampled to characterise how infrequent a connected component of order at least *r* is when *k* nodes are uniformly sampled. The assumption behind this filter is that meaningful inputs encompass metabolites relatively close to each other within the knowledge graph, prone to be reported in large connected components involving most of them.

### Classes

*FELLA* relies on two classes: *FELLA.DATA* for the internal knowledge representation, based on the *igraph* R package [13], and *FELLA.USER* for the user analysis, see Fig. 1. These classes contain subclasses, invisible to the user and described in the Additional file 2. The functions to manipulate both classes are described below, following the three blocks from Fig. 1.

#### Block I: local database

The function buildGraphFromKEGGREST() retrieves the tabular KEGG data for the desired organism and builds the knowledge graph as described in [12]. Then, a database can be built from the graph and stored in a local folder using buildDataFromGraph(). Databases are needed for the enrichment and should be loaded through the function loadKEGGdata().

#### Block II: enrichment analysis

Once the database is loaded, i.e. the *FELLA.DATA* object is in memory, defineCompounds() maps the list of input metabolites, in the form of KEGG identifiers, to the internal representation, providing a *FELLA.USER* object. Then, the propagation algorithms in [12] are run to score the graph nodes. runDiffusion() uses the undirected heat diffusion model [10] whereas runPagerank() runs the directed PageRank algorithm [11]. Both approaches are automatically followed by the statistical normalisation, either as a parametric z-score (approx = "normality") or as a simulated permutation analysis (approx = "simulation"), see Table 1. The wrapper enrich() performs the metabolite mapping and the desired propagation algorithm (argument method) and statistical normalisation with a single call.

**Table 1:** Scoring methods offered in *FELLA*, chosen by the enrich function arguments method and approx

Method	Approx	Notation in [12]	Comment
Hypergeom	N/A	Hypergeometric test	Included for reference
Diffusion	Normality	HD norm	Heat diffusion scores followed by z-scores
Diffusion	Simulation	HD sim	Heat diffusion scores followed by permutations
Pagerank	Normality	PR norm	PageRank scores followed by z-scores
Pagerank	Simulation	PR sim	PageRank scores followed by permutations

Each row corresponds to a method mentioned in the original publication [12]. The method hypergeom is Fisher’s exact test, included for reference. Method diffusion scores the nodes using the heat diffusion model. Method pagerank uses the PageRank algorithm on an upwards-directed version of the network. Both scores undergo a statistical normalisation to remove structural biases, controlled through the approx argument. The user can choose the fast, parametric z-scores (normality) or the slower, non-parametric permutation analysis (simulation). N/A: non-applicable

#### Block III: exporting results

Finally, the best scoring KEGG entries can be visualised through plot(), exported as a sub-network with generateResultsGraph(), or in tabular format with generateResultsTable(). A dedicated table with the reported enzymes and its associated genes can be obtained with generateEnzymesTable(). Alternatively, exportResults() allows writing such objects directly to files.

### User interface

**Fig. 3:**
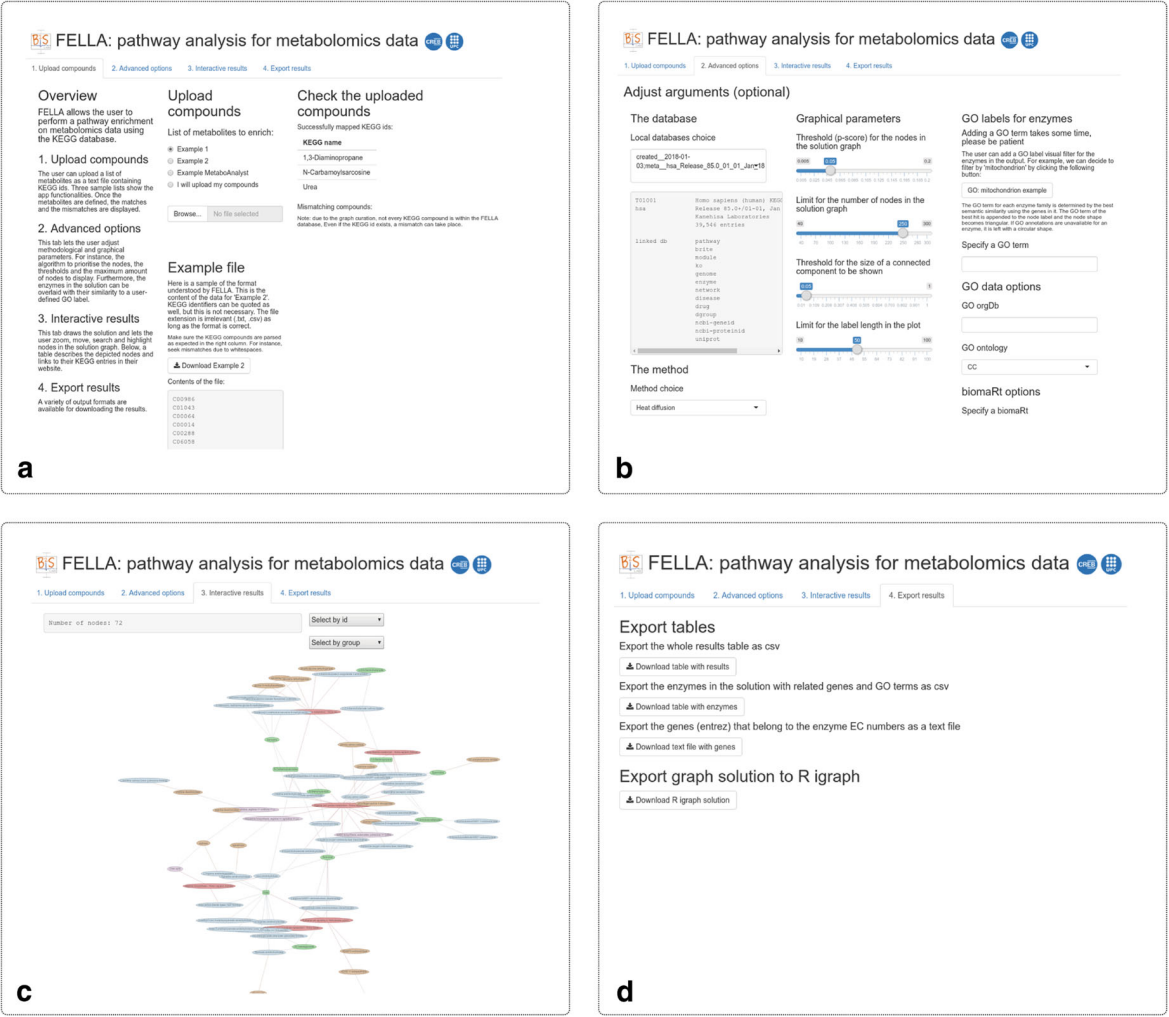
Perspective of the interactive app within *FELLA*. The app is composed by four tabs: **a** compounds upload, **b** advanced options, **c** results and **d** export. The lay user can rapidly explore his or her data without knowing the details about the syntax in *FELLA*

*FELLA* includes an interactive graphical interface, based on the R package *shiny* [14] and deployable through launchApp(). The interface is divided with four tabs that encompass most options from *FELLA* (Fig. 3). Currently, the database needs to be built outside the graphical interface and prior to its usage.

#### Compounds upload

This tab contains a general description of the interface and a handle to submit the input metabolite list as a text file. Examples are provided as well. The right panel shows the mapped and the mismatching compounds with regard to the current database.

#### Advanced options

Widgets from this tab adjust the main function arguments for customising the enrichment procedure. They ease database choice from the internal package directory, method and approximation definition and parameter tweaking. It also allows the semantic similarity analysis on the reported enzymes, using the R package *GOSemSim* [15] with the Gene Ontology annotations [16].

#### Results and discussion

The results section mainly consists of an interactive network plot with the top *k* KEGG entries. Nodes can be moved, selected, queried and hovered to reveal the original KEGG entry. An interactive table lies below the plot and expands the data on the nodes.

#### Export

The last tab offers several options to download the reported sub-network (tabular format or R object) and enzymes (tabular format).

## Results

The algorithmic part of *FELLA* has already been discussed and validated in [12]. The usage of *FELLA* is hereby demonstrated on three public human studies on epithelial cells [17], ovarian cancer cells [18] and febrile illnesses [19]. The examples guide the user on how to build the database, format the input data, complete the enrichment and export its results (see Additional file 2). *FELLA* reproduces findings from the original publications, not only in the form of pathway hits but also as newly suggested enzymes and metabolites. The Additional file 5 shows further details on the metabolites in each input and the reported sub-networks.

To demonstrate its usefulness outside human studies, *FELLA* is applied to two datasets from a gilt-head bream study [20] and a mouse model of non-alcoholic fatty liver disease [21]. The complete analyses can be respectively found in Additional files 3 and 4, whereas their respective R workspaces are saved in Additional files 6 and 7. Table 2 summarises the knowledge graphs in the *FELLA.DATA* object for each organism.

**Table 2:** Summary of the *FELLA.DATA* objects used for the three human and the three non-human datasets

Organism	KEGG release	Nodes	Pathways	Modules	Enzymes	Reactions	Compounds
*Homo sapiens*	85.0+/02-16	9899	314	182	1110	4829	3464
*Danio rerio*	87.0+/09-14	9637	162	179	995	4843	3458
*Mus musculus*	87.0+/09-14	9909	316	185	1107	4843	3458

Generalist and overview pathways are excluded from the models, see Additional files 2, 3 and 4 for further details on each organism

### Epithelial cells dataset

**Fig. 4:**
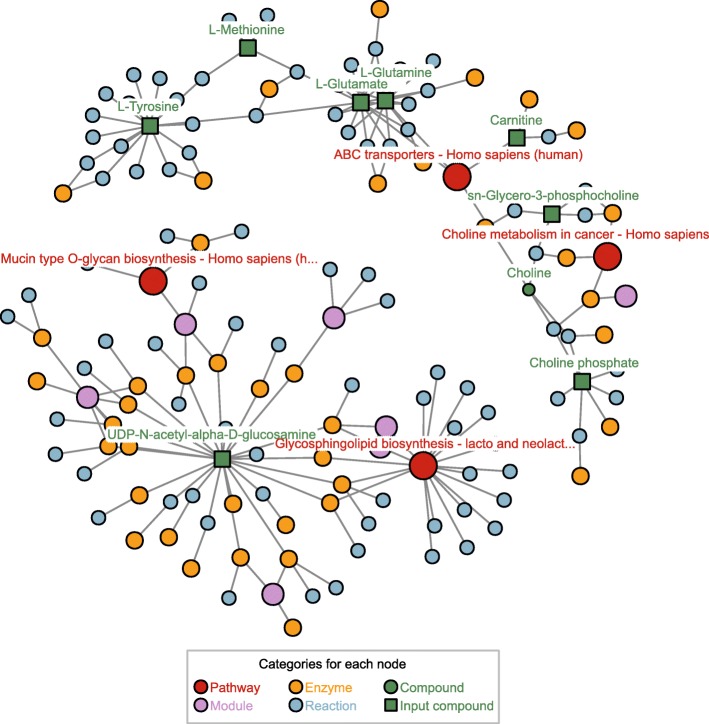
Results of the node prioritisation by *FELLA* in the epithelial cells dataset. The user is given a list of positive entities, after a score threshold described in [12], with information on how the input metabolites reach the suggested pathways and on how these pathways cross talk. Plots of the ovarian and malaria datasets can be found in the Additional file 5

The epithelial cancer cells study [17] runs an in vitro model of dry eye in which the human epithelial cells IOBA-NHC are put under hyperosmotic stress. The list of 9 metabolites hereby used reflects metabolic changes in “Treatment 1” (24 h in serum-free media at 380 mOsm) against control (24 h at 280 mOsm). The metabolites have been extracted from “Table 1” in the original manuscript and mapped to 9 KEGG ids, from which 8 map to the *FELLA.DATA* object. The enrichment (sub-network in Fig. 4) is obtained by leaving the default parameters in *FELLA*: method = "diffusion", approx = "normality" and threshold = 0.05. The amount of nodes has been limited to nlimit = 150.

The activation of the “glycerophosphocholine synthesis” rather than the “carnitine” response is a main result in the original work [17]. *FELLA* highlights the related pathway “choline metabolism in cancer” and the “choline” metabolite as well. Another key process is the “O-linked glycosilation”, which is close to the KEGG module “O-glycan biosynthesis, mucin type core” and to the KEGG pathway “Mucin type O-glycan biosynthesis”. Finally, *FELLA* reproduces the finding of “UAP1” by reporting the enzyme “2.7.7.23”, named “UDP-N-acetylglucosamine diphosphorylase”. “UAP1” is a key protein in the study, pinpointed by iTRAQ (Isobaric Tags for Relative and Absolute Quantitation) and validated via western blot.

### Ovarian cancer cells dataset

The second dataset has been extracted from the study on metabolic responses of ovarian cancer cells [18]. OCSCs are isogenic ovarian cancer stem cells derived from the OVCAR-3 ovarian cancer cells. The abundances of 6 metabolites are affected by the exposure to several environmental conditions: glucose deprivation, hypoxia and ischemia. From those, 5 metabolites map to the *FELLA.DATA* object. The sub-network is obtained by leaving the default parameters and setting a limit of nlimit = 150 nodes.

Several “TCA cycle”-related entities are highlighted, also found by the authors and by previous work [22]. It also mentions “sphingosine degradation”, closely related to the reported “sphingosine metabolism” in the original work. Enzymes that have been formerly related to cancer are suggested within the TCA cycle, like “fumarate hydratase” [22–24], “succinate dehydrogenase” [22, 25] and “aconitase” [26]. Another suggestion is “lysosome”(s), known to suffer changes in cancer cells and directly affect apoptosis [27]. Finally, the graph contains several “hexokinases”, potential targets to disrupt glycolysis, a fundamental need in cancer cells [28].

### Malaria dataset

The metabolites in this example are related to the distinction between malaria and other febrile illnesses [19]. Specifically, the list of 11 KEGG identifiers (9 in the *FELLA.DATA* object) has been extracted from the original supplementary data spreadsheet, using all the possible KEGG matches for the “non malaria” patient group. The sub-network is obtained by leaving the default parameters and setting a limit of nlimit = 50 nodes.

In this case, the depicted subnetwork contains the modules “C21-Steroid hormone biosynthesis, progesterone =>corticosterone/aldosterone” and “C21-Steroid hormone biosynthesis, progesterone =>cortisol/cortisone”, related to the “corticosteroids” as a main pathway reported in the original text. This is part of the also reported “Aldosterone synthesis and secretion”; aldosterone is known to show changes related to fever as a metabolic response to infection [29]. Another plausible hit in the sub-network is “linoleic acid metabolism”, as erythrocytes infected by various malaria parasytes can be enriched in linoleic acid [30]. In addition, the pathway “sphingolipid metabolism” can play a role in the immune response [31, 32]. As for the enzymes, “3alpha-hydroxysteroid 3-dehydrogenase (Si-specific)” and “Delta4-3-oxosteroid 5beta-reductase” are related to three input metabolites each and might be candidates for further examination.

### Oxybenzone exposition on gilt-head bream datasets

A study of the consequences of the oxybenzone contaminant on gilt-head bream [20] found five dysregulated KEGG metabolites in their liver and eleven in their plasma. The study justified its findings through literature and complemented them with insights provided by *FELLA*. Here, both metabolite lists are used to build suggested sub-networks with the default parameters and fixing nlimit = 250. The *FELLA.DATA* object is built for the *Danio Rerio* organism, a common approximation when annotations specific to gilt-head bream are not available. Further details can be found in the vignette (Additional file 3) and its workspace (Additional file 6).

The enrichment on the liver-derived metabolites links all of them within a connected component of roughly 100 nodes. It points to “Phenylalanine metabolism” as one of the key metabolic pathways, in accordance with the main results from the article. Among the suggested metabolites, “Tyrosine” is of particular help to explain the connection between the affected metabolites (see Fig. 2 from [20]).

Plasma metabolites involve a more complex scenario. *FELLA* reports ten out of the eleven metabolites in a connected component involving around 120 nodes. Seven pathways are suggested, from which “Linoleic acid metabolism”, “Biosynthesis of unsaturated fatty acids”, “alpha-Linolenic acid metabolism”, “Glycerophospholipid metabolism” and “Glycine, serine and threonine metabolism” were used to build a comprehensive picture of the metabolic changes in the original manuscript (Fig. 3 from [20]). Such figure brings a structured overview that narrows down the core processes, also backed up by prior publications. Likewise, by drawing intermediate metabolites found through *FELLA*, like “Linoleic acid” and “Phosphatidylcholine”, it achieves a cohesive representation of the input metabolites.

### Non-alcoholic fatty liver disease mouse model

This dataset exemplifies how *FELLA* can also be applied on an animal disease model. Metabolites in liver tissue from leptin-deficient *ob/ob* mice and wild-type were compared using Nuclear Magnetic Resonance, whereas several candidate genes were further investigated for differences in expression [21]. Six affected metabolites are introduced in *FELLA*, leaving the default parameters and nlimit = 250. The *FELLA.DATA* object is built for the *Mus musculus* organism. The vignette with the whole analysis is provided provided as Additional file 4, whereas its R workspace can be found in Additional file 7.

The sub-network found by *FELLA* involves “N,N-Dimethylglycine”, a marginally significant metabolite in the experimental data but with a relevant role within the findings from the study. Regarding the genes, *FELLA* is able to find the enzyme associated to *Bhmt*, validated and discussed in the study. The enzyme associated to *Cbs*, another central hit, is not directly found. However, its ranking (top 17% among enzymes) and especially that of its reaction (top 3% among reactions) are highly suggestive. We also show how other (1) related metabolites, found by leveraging the expression data, and (2) differentially expressed genes, taken from an external study [33], tend to have top p-scores in the prioritisation provided by *FELLA*.

## Conclusions

We present *FELLA*, an R package for enriching metabolomics data, focused on interpretability. It can be used either programmatically or through a simple user interface. *FELLA* offers a comprehensive enrichment by depicting the intermediate reactions, enzymes and modules that link the input metabolites to the relevant pathways. This layout gives a biological picture with information of the pathway overlap and the connections between the entities of interest, while suggesting enzymes and possibly other metabolites for further study. The utility of *FELLA* has been demonstrated on six public datasets, both with human and non-human organisms, where reported entities include several original findings in addition to results from third studies. *FELLA* is publicly available in the Bioconductor public repository under the GPL-3 license.

## Availability and requirements

**Project name**: FELLA

**Project home page**: https://doi.org/doi:10.18129/B9.bioc.FELLA, https://github.com/b2slab/FELLA

**Operating system(s)**: platform independent

**Programming language**: R

**Other requirements**: none

**License**: GPL-3

**Restrictions to use by non-academics**: those derived by the GPL-3 license

## Additional files

Additional file 1 User guide within *FELLA* showing fast and concise toy examples of its application. (HTML 2587 kb)

Additional file 2 User guide within the R package *FELLA* with background, implementation details and three real examples on its usage. (PDF 1096 kb)

Additional file 3 Case study with *FELLA*: two datasets on the effect of oxybenzone exposition on gilt-head bream. (PDF 221 kb)

Additional file 4 Case study with *FELLA*: a multi-omic mouse model of non-alcoholic fatty liver disease. (PDF 235 kb)

Additional file 5 Descriptive files on the three human datasets: a summary of the inputs (descriptive_input.csv), input and reported subgraph in each dataset (dataset_input.csv, dataset_subgraph.csv and dataset_subgraph.pdf), hits discussed in the results section (descriptive_hits.csv). Also contains the database object (fella_data.RData) and metadata about the database (info_fella_data.txt), the KEGG version (info_kegg.txt) and the R session (info_session.txt). (ZIP 525 kb)

Additional file 6 R workspace from the gilt-head bream datasets. (ZIP 590 kb)

Additional file 7 R workspace from the mouse model study. (ZIP 829 kb)

## Abbreviations

FCS: Functional class scoring
GPL-3: General public license version 3
iTRAQ: Isobaric tags for relative and absolute quantitation
KEGG: Kyoto encyclopedia of genes and genomes
N/A: Non-applicable
ORA: Over representation analysis
PT: Pathway topology-based
TCA: TriCarboxylic acid
UDP: Uridine diphosphate
