# Supplementary material for: FELLA: an R package to enrich metabolomics data
Source: BMC Bioinformatics. 2018 Dec 22;19:538. doi: 10.1186/s12859-018-2487-5 (PMC6303911; doi:10.1186/s12859-018-2487-5)
Supplement: Supplementary file 1 — User guide within FELLA showing fast and concise toy examples of its application. (HTML 2587 kb) [file 12859_2018_2487_MOESM1_ESM.html]

An overview of FELLA: data enrichment for metabolomics summary data


# An overview of FELLA: data enrichment for metabolomics summary data

Sergio Picart-Armada1\* and Alexandre Perera-Lluna1\*\*

1B2SLab at Polytechnic University of Catalonia

\*sergi.picart@upc.edu  
\*\*alexandre.perera@upc.edu

#### *21 February 2018*

#### Package

FELLA 0.99.3.1

# Contents

- 1 Introduction
- 2 Loading the KEGG data
- 3 Loading the metabolomics summary data
- 4 Enriching the data
  - 4.1 Enrichment methods
  - 4.2 Statistical approximations
  - 4.3 Enrichment: methods, approximations and wrapper function
- 5 Visualising the results
  - 5.1 Hypergeom
  - 5.2 Diffusion
  - 5.3 PageRank
- 6 Exporting the results
  - 6.1 Exporting inside R
  - 6.2 Exporting outside R
- 7 Session info

# 1 Introduction

`FELLA` is an R package that brings a new concept for metabolomics data interpretation. The starting point of this data enrichment is a list of affected metabolites, which can stem from a contrast between experimental groups. This list, that may vary in size, encompasses key role players from different **biological pathways** that generate a biological perturbation.

The classical way to analyse this list is the **over representation analysis**. Each metabolic pathway has a statistic, the number of affected metabolites in it, that yields a p-value. After correcting for multiple testing, a list of prioritised pathways helps performing a quality check on the data and suggesting novel biological mechanisms related to the data. Subsequent generations of **pathway analysis** methods attempt to include quantitative and/or topological data in the statistics in order to improve power for subtle signals, but the interpretation of a prioritised pathway list remains a challenge.

Package `FELLA`, on the other hand, introduces a comprehensive output that encompasses other biological entities that coherently relate the top ranked pathways. The priorisation of the pathways and other entiteis stems from a diffusion process on a holistic **graph representation** of the **KEGG database**. `FELLA` needs:

1. The KEGG graph and other complementary data files. This is stored in a unique `FELLA.DATA` S4 object.
2. A list of affected metabolites (KEGG compounds). This is stored in a unique `FELLA.USER` S4 object, along with user analyses.

# 2 Loading the KEGG data

This vignette makes use of sample data that contains small subgraph of `FELLA`’s KEGG graph (mid 2017 KEGG release). All the necessary contextual data is stored in an S4 data structure with class `FELLA.DATA`. Several functions need access to the contextual data, passed as an argument called `data`, being the enrichment itself among them.

```
library(FELLA)

data("FELLA.sample")
class(FELLA.sample)
```

```
## [1] "FELLA.DATA"
## attr(,"package")
## [1] "FELLA"
```

```
show(FELLA.sample)
```

```
## General data:
## - KEGG graph:
##   * Nodes:  670 
##   * Edges:  1677 
##   * Density:  0.003741383 
##   * Categories:
##     + pathway [2]
##     + module [6]
##     + enzyme [58]
##     + reaction [279]
##     + compound [325]
##   * Size:  366.9 Kb 
## - KEGG names are ready.
## -----------------------------
## Hypergeometric test:
## - Matrix is ready
##   * Dim:  325 x 2 
##   * Size:  25 Kb
## -----------------------------
## Heat diffusion:
## - Matrix not loaded.
## - RowSums are ready.
## -----------------------------
## PageRank:
## - Matrix not loaded.
## - RowSums are ready.
```

Keep in mind that `FELLA.DATA` objects need to be constructed only once by using `buildGraphFromKEGGREST` and `buildDataFromGraph`, in that precise order. This will store them in a local path and they should be loaded through `loadKEGGdata`. The user is disadvised from manually modifying the database internal files and the `FELLA.DATA` object slots not to corrupt the database.

# 3 Loading the metabolomics summary data

The second block of necessary data is a list of affected metabolites, which shoud be specified as KEGG compound IDs. Provided is a list of hypothetical affected metabolites belonging to the graph, to which some decoys that do not map to the graph are added.

```
data("input.sample")
input.full <- c(input.sample, paste0("intruder", 1:10))

show(input.full)
```

```
##  [1] "C00143"     "C00546"     "C04225"     "C16328"     "C00091"    
##  [6] "C15979"     "C16333"     "C05264"     "C05258"     "C00011"    
## [11] "C00083"     "C00044"     "C05266"     "C00479"     "C05280"    
## [16] "C01352"     "C05268"     "C16329"     "C00334"     "C05275"    
## [21] "C14145"     "C00081"     "C04253"     "C00027"     "C00111"    
## [26] "C00332"     "C00003"     "C00288"     "C05467"     "C00164"    
## [31] "intruder1"  "intruder2"  "intruder3"  "intruder4"  "intruder5" 
## [36] "intruder6"  "intruder7"  "intruder8"  "intruder9"  "intruder10"
```

Compounds are introduced through the `defineCompounds` function and provide the first `FELLA.USER` user data object containing the mapped compounds and empty analyses slots. The user should always build `FELLA.USER` objects through `defineCompounds` instead of manipulating the slots of the object manually - this might skip quality checks.

```
myAnalysis <- defineCompounds(
    compounds = input.full, 
    data = FELLA.sample)
```

```
## No background compounds specified. Default background will be used.
```

```
## Warning in defineCompounds(compounds = input.full, data = FELLA.sample):
## Some compounds were introduced as affected but they do not belong to
## the background. These compounds will be excluded from the analysis. Use
## 'getExcluded' to see them.
```

Note that a warning message informs the user that some compounds did not map to the KEGG compound collection. Compounds that successfully mapped can be obtained through `getInput`,

```
getInput(myAnalysis)
```

```
##  [1] "C00003" "C00011" "C00027" "C00044" "C00081" "C00083" "C00091" "C00111"
##  [9] "C00143" "C00164" "C00288" "C00332" "C00334" "C00479" "C00546" "C01352"
## [17] "C04225" "C04253" "C05258" "C05264" "C05266" "C05268" "C05275" "C05280"
## [25] "C05467" "C14145" "C15979" "C16328" "C16329" "C16333"
```

while compounds that were excluded because of mismatch can be accessed through `getExcluded`:

```
getExcluded(myAnalysis)
```

```
##  [1] "intruder1"  "intruder2"  "intruder3"  "intruder4"  "intruder5" 
##  [6] "intruder6"  "intruder7"  "intruder8"  "intruder9"  "intruder10"
```

Keep in mind that exact matching is sought, so be extremely careful with **whitespaces**, tabs or similar characters that might create mismatches. For example:

```
input.fail <- paste0(" ", input.full)
defineCompounds(
    compounds = input.fail, 
    data = FELLA.sample)
```

```
## Error in defineCompounds(compounds = input.fail, data = FELLA.sample): None of the specified compounds appear in the available KEGG data.
```

# 4 Enriching the data

Once the `FELLA.DATA` and the `FELLA.USER` with the affected metabolites are ready, the data can be easily enriched.

## 4.1 Enrichment methods

There are three methods to enrich:

1. **Hypergeometric test** (`method = "hypergeom"`): it performs the metabolite-sampling hypergeometric test using the connections in `FELLA`’s KEGG graph. This is included for completeness and does not include the contextual novelty of the diffusive methods.
2. **Diffusion** (`method = "diffusion"`): it performs sub-network analysis on the KEGG graph to extract a meaningful subgraph. This subgraph can be plotted an interpreted
3. **PageRank** (`method = "pagerank"`): analogous to `"diffusion"` but using the directed diffusion, which matches the PageRank algorithm for web ranking.

## 4.2 Statistical approximations

For methods `"diffusion"` and `"pagerank"`, two statistical approximations are proposed:

1. **Normal approximation** (`approx = "normality"`): scores are computed through z-scores based on analytical expected value and covariance matrix of the null model for diffusion. This approximation is deterministic and fast.
2. **Monte Carlo trials** (`approx = "simulation"`): scores are computed through Monte Carlo trials of the random variables. This approximation requires computing the random trials, governed by the `ntrials` argument.

## 4.3 Enrichment: methods, approximations and wrapper function

The function `enrich` wraps the functions `defineCompounds`, `runHypergeom`, `runDiffusion` and `runPagerank` in an easily usable manner, returning a `FELLA.USER` object with complete analyses.

```
myAnalysis <- enrich(
    compounds = input.full, 
    method = "diffusion", 
    approx = "normality", 
    data = FELLA.sample)
```

```
## No background compounds specified. Default background will be used.
```

```
## Warning in defineCompounds(compounds = compounds, compoundsBackground =
## compoundsBackground, : Some compounds were introduced as affected but they
## do not belong to the background. These compounds will be excluded from the
## analysis. Use 'getExcluded' to see them.
```

```
## Running diffusion...
```

```
## Computing p-scores through the specified distribution.
```

```
## Done.
```

The output is quite informative and aggregates all the warnings. Let’s compare an empty `FELLA.USER` object

```
show(new("FELLA.USER"))
```

```
## Compounds in the input: empty
## Background compounds: all available compounds (default)
## -----------------------------
## Hypergeometric test: not performed
## -----------------------------
## Heat diffusion: not performed
## -----------------------------
## PageRank: not performed
```

to the output of a processed one:

```
show(myAnalysis)
```

```
## Compounds in the input: 30
##  [1] "C00003" "C00011" "C00027" "C00044" "C00081" "C00083" "C00091" "C00111"
##  [9] "C00143" "C00164" "C00288" "C00332" "C00334" "C00479" "C00546" "C01352"
## [17] "C04225" "C04253" "C05258" "C05264" "C05266" "C05268" "C05275" "C05280"
## [25] "C05467" "C14145" "C15979" "C16328" "C16329" "C16333"
## Background compounds: all available compounds (default)
## -----------------------------
## Hypergeometric test: not performed
## -----------------------------
## Heat diffusion: ready.
## P-scores under 0.05:  86
## -----------------------------
## PageRank: not performed
```

The wrapper function `enrich` can run the three analysis at once with the option `method = listMethods()`, or only the desired ones providing them as a character vector:

```
myAnalysis <- enrich(
    compounds = input.full, 
    method = listMethods(), 
    approx = "normality", 
    data = FELLA.sample)

show(myAnalysis)
```

```
## Compounds in the input: 30
##  [1] "C00003" "C00011" "C00027" "C00044" "C00081" "C00083" "C00091" "C00111"
##  [9] "C00143" "C00164" "C00288" "C00332" "C00334" "C00479" "C00546" "C01352"
## [17] "C04225" "C04253" "C05258" "C05264" "C05266" "C05268" "C05275" "C05280"
## [25] "C05467" "C14145" "C15979" "C16328" "C16329" "C16333"
## Background compounds: all available compounds (default)
## -----------------------------
## Hypergeometric test: ready.
## Top 2 p-values:
##     hsa00640     hsa00010 
## 8.540386e-09 9.999888e-01 
## 
## -----------------------------
## Heat diffusion: ready.
## P-scores under 0.05:  86
## -----------------------------
## PageRank: ready.
## P-scores under 0.05:  70
```

The wrapped functions work in a similar way, here is an example with `runDiffusion`:

```
myAnalysis_bis <- runDiffusion(
    object = myAnalysis, 
    approx = "normality", 
    data = FELLA.sample)
```

```
## Running diffusion...
```

```
## Computing p-scores through the specified distribution.
```

```
## Done.
```

```
show(myAnalysis_bis)
```

```
## Compounds in the input: 30
##  [1] "C00003" "C00011" "C00027" "C00044" "C00081" "C00083" "C00091" "C00111"
##  [9] "C00143" "C00164" "C00288" "C00332" "C00334" "C00479" "C00546" "C01352"
## [17] "C04225" "C04253" "C05258" "C05264" "C05266" "C05268" "C05275" "C05280"
## [25] "C05467" "C14145" "C15979" "C16328" "C16329" "C16333"
## Background compounds: all available compounds (default)
## -----------------------------
## Hypergeometric test: ready.
## Top 2 p-values:
##     hsa00640     hsa00010 
## 8.540386e-09 9.999888e-01 
## 
## -----------------------------
## Heat diffusion: ready.
## P-scores under 0.05:  86
## -----------------------------
## PageRank: ready.
## P-scores under 0.05:  70
```

# 5 Visualising the results

The method `plot` for data from the package `FELLA` allows a friendly visualisation of the relevant part of the KEGG graph.

## 5.1 Hypergeom

In the case `method = "hypergeom"` the plot encompasses a bipartite graph that contains top pathways and affected compounds. In that case, `threshold = 1` allows the visualisation of both pathways; otherwise a plot with only one pathway would be quite uninformative.

```
plot(
    x = myAnalysis, 
    method = "hypergeom", 
    main = "My first enrichment using the hypergeometric test in FELLA", 
    threshold = 1, 
    data = FELLA.sample)
```

## 5.2 Diffusion

For `method = "diffusion"` the graph contains a richer representations involving **modules, enzymes and reactions** that link affected pathways and compounds.

```
plot(
    x = myAnalysis, 
    method = "diffusion", 
    main = "My first enrichment using the diffusion analysis in FELLA", 
    threshold = 0.1, 
    data = FELLA.sample)
```

## 5.3 PageRank

For `method = "pagerank"` the concept is analogous to diffusion:

```
plot(
    x = myAnalysis, 
    method = "pagerank", 
    main = "My first enrichment using the PageRank analysis in FELLA", 
    threshold = 0.1, 
    data = FELLA.sample)
```

# 6 Exporting the results

`FELLA` offers several exporting alternatives, both for the R environment and for external software.

## 6.1 Exporting inside R

The appropriate functions to export the results inside R are `generateResultsTable` for a **data.frame** object:

```
myTable <- generateResultsTable(
    object = myAnalysis, 
    method = "diffusion", 
    threshold = 0.1, 
    data = FELLA.sample)
```

```
## Writing diffusion results...
```

```
## Done.
```

```
knitr::kable(head(myTable, 20))
```

| KEGG.id | Entry.type | KEGG.name | p.score |
| --- | --- | --- | --- |
| hsa00640 | pathway | Propanoate metabolism - Homo sapiens (human) | 0.0036894 |
| M00013 | module | Malonate semialdehyde pathway, propanoyl-CoA … | 0.0044683 |
| 1.1.1.211 | enzyme | long-chain-3-hydroxyacyl-CoA dehydrogenase | 0.0371099 |
| 1.1.1.35 | enzyme | 3-hydroxyacyl-CoA dehydrogenase | 0.0392511 |
| 1.2.1.18 | enzyme | malonate-semialdehyde dehydrogenase (acetylat… | 0.0069255 |
| 1.2.1.27 | enzyme | methylmalonate-semialdehyde dehydrogenase (Co… | 0.0165439 |
| 2.3.1.9 | enzyme | acetyl-CoA C-acetyltransferase | 0.0085923 |
| 3.1.2.4 | enzyme | 3-hydroxyisobutyryl-CoA hydrolase | 0.0786804 |
| 4.1.1.32 | enzyme | phosphoenolpyruvate carboxykinase (GTP) | 0.0700429 |
| 4.1.1.41 | enzyme | (S)-methylmalonyl-CoA decarboxylase | 0.0223899 |
| 4.1.1.9 | enzyme | malonyl-CoA decarboxylase | 0.0002538 |
| 4.2.1.17 | enzyme | enoyl-CoA hydratase | 0.0015731 |
| 5.3.3.8 | enzyme | dodecenoyl-CoA isomerase | 0.0164255 |
| 6.2.1.4 | enzyme | succinate—CoA ligase (GDP-forming) | 0.0019142 |
| 6.2.1.5 | enzyme | succinate—CoA ligase (ADP-forming) | 0.0125330 |
| R00209 | reaction | pyruvate:NAD+ 2-oxidoreductase (CoA-acetylati… | 0.0885938 |
| R00233 | reaction | malonyl-CoA carboxy-lyase (acetyl-CoA-forming… | 0.0000698 |
| R00238 | reaction | Acetyl-CoA:acetyl-CoA C-acetyltransferase | 0.0001037 |
| R00353 | reaction | malonyl-CoA:pyruvate carboxytransferase | 0.0065794 |
| R00405 | reaction | Succinate:CoA ligase (ADP-forming) | 0.0468613 |

…and `generateResultsGraph` for a **graph** in igraph format:

```
myGraph <- generateResultsGraph(
    object = myAnalysis, 
    method = "diffusion", 
    threshold = 0.1, 
    data = FELLA.sample)

show(myGraph)
```

```
## IGRAPH b25c0a7 UNW- 102 166 -- 
## + attr: name (v/c), com (v/n), NAME (v/x), entrez (v/x), label
## | (v/c), input (v/l), weight (e/n)
## + edges from b25c0a7 (vertex names):
##  [1] hsa00640--M00013    M00013  --1.1.1.211 M00013  --1.1.1.35 
##  [4] M00013  --1.2.1.18  M00013  --1.2.1.27  hsa00640--2.3.1.9  
##  [7] M00013  --3.1.2.4   hsa00640--4.1.1.41  hsa00640--4.1.1.9  
## [10] M00013  --4.2.1.17  M00013  --5.3.3.8   hsa00640--6.2.1.4  
## [13] hsa00640--6.2.1.5   4.1.1.9 --R00233    2.3.1.9 --R00238   
## [16] hsa00640--R00353    6.2.1.5 --R00405    4.1.1.32--R00431   
## [19] 6.2.1.4 --R00432    1.2.1.18--R00705    1.2.1.27--R00705   
## + ... omitted several edges
```

## 6.2 Exporting outside R

Results can be saved as permanent files. The **data.frame** data format can be saved as a `.csv` file:

```
myTempDir <- tempdir()
myExp_csv <- paste0(myTempDir, "/table.csv")
exportResults(
    format = "csv", 
    file = myExp_csv, 
    method = "pagerank", 
    threshold = 0.1, 
    object = myAnalysis, 
    data = FELLA.sample)
```

```
## Exporting to a csv file...
```

```
## Writing pagerank results...
```

```
## Done.
```

```
## Done
```

```
test <- read.csv(file = myExp_csv)
knitr::kable(head(test))
```

| KEGG.id | Entry.type | KEGG.name | p.score |
| --- | --- | --- | --- |
| hsa00640 | pathway | Propanoate metabolism - Homo sapiens (human) | 0.0000085 |
| M00013 | module | Malonate semialdehyde pathway, propanoyl-CoA … | 0.0010330 |
| 1.1.1.35 | enzyme | 3-hydroxyacyl-CoA dehydrogenase | 0.0422528 |
| 4.1.1.32 | enzyme | phosphoenolpyruvate carboxykinase (GTP) | 0.0088747 |
| 4.1.1.9 | enzyme | malonyl-CoA decarboxylase | 0.0005280 |
| 4.2.1.17 | enzyme | enoyl-CoA hydratase | 0.0003343 |

In the same line, the **graph** can be saved in `RData`:

```
myExp_graph <- paste0(myTempDir, "/graph.RData")
exportResults(
    format = "igraph", 
    file = myExp_graph, 
    method = "pagerank", 
    threshold = 0.1, 
    object = myAnalysis, 
    data = FELLA.sample)
```

```
## Exporting to a RData file using 'igraph' object...
```

```
## Done
```

```
stopifnot("graph.RData" %in% list.files(myTempDir))
```

Other formats exported by igraph are also available, internally using their function `igraph::write.graph`. Check the **format** argument of `?igraph::write.graph` for a list of the supported formats. For example, using `"pajek"` format:

```
myExp_pajek <- paste0(myTempDir, "/graph.pajek")
exportResults(
    format = "pajek", 
    file = myExp_pajek, 
    method = "diffusion", 
    threshold = 0.1, 
    object = myAnalysis, 
    data = FELLA.sample)
```

```
## Exporting to the format pajek using igraph...
```

```
## Done
```

```
stopifnot("graph.pajek" %in% list.files(myTempDir))
```

This option is toggled if the format does not match any other predefined export option.

# 7 Session info

For reproducibility purposes, below is the `sessionInfo()` output:

```
sessionInfo()
```

```
## R Under development (unstable) (2017-12-31 r73996)
## Platform: x86_64-pc-linux-gnu (64-bit)
## Running under: Debian GNU/Linux 9 (stretch)
## 
## Matrix products: default
## BLAS: /usr/lib/openblas-base/libblas.so.3
## LAPACK: /usr/lib/libopenblasp-r0.2.19.so
## 
## locale:
##  [1] LC_CTYPE=en_US.UTF-8       LC_NUMERIC=C              
##  [3] LC_TIME=en_US.UTF-8        LC_COLLATE=en_US.UTF-8    
##  [5] LC_MONETARY=en_US.UTF-8    LC_MESSAGES=C             
##  [7] LC_PAPER=en_US.UTF-8       LC_NAME=C                 
##  [9] LC_ADDRESS=C               LC_TELEPHONE=C            
## [11] LC_MEASUREMENT=en_US.UTF-8 LC_IDENTIFICATION=C       
## 
## attached base packages:
## [1] stats     graphics  grDevices utils     datasets  methods   base     
## 
## other attached packages:
##  [1] FELLA_0.99.3.1            BiocStyle_2.7.8          
##  [3] diffuStats_0.103.2        precrec_0.9.1            
##  [5] plyr_1.8.4                RcppParallel_4.3.20      
##  [7] RcppArmadillo_0.8.300.1.0 Rcpp_0.12.14             
##  [9] igraph_1.1.2              expm_0.999-2             
## [11] MASS_7.3-48               Matrix_1.2-12            
## 
## loaded via a namespace (and not attached):
##  [1] highr_0.6            XVector_0.19.8       pillar_1.0.1        
##  [4] compiler_3.5.0       BiocInstaller_1.29.4 zlibbioc_1.25.0     
##  [7] tools_3.5.0          digest_0.6.15        memoise_1.1.0       
## [10] evaluate_0.10.1      tibble_1.4.1         gtable_0.2.0        
## [13] lattice_0.20-35      png_0.1-7            pkgconfig_2.0.1     
## [16] rlang_0.2.0          parallel_3.5.0       yaml_2.1.16         
## [19] xfun_0.1             httr_1.3.1           withr_2.1.1         
## [22] stringr_1.3.0        knitr_1.20           IRanges_2.13.26     
## [25] S4Vectors_0.17.33    Biostrings_2.47.9    devtools_1.13.4     
## [28] stats4_3.5.0         rprojroot_1.3-1      grid_3.5.0          
## [31] data.table_1.10.4-3  R6_2.2.2             rmarkdown_1.8       
## [34] bookdown_0.6         ggplot2_2.2.1        magrittr_1.5        
## [37] BiocGenerics_0.25.3  scales_0.5.0         backports_1.1.2     
## [40] htmltools_0.3.6      KEGGREST_1.19.2      colorspace_1.3-2    
## [43] stringi_1.1.6        lazyeval_0.2.1       munsell_0.4.3
```
