# Supplementary material for: FELLA: an R package to enrich metabolomics data
Source: BMC Bioinformatics. 2018 Dec 22;19:538. doi: 10.1186/s12859-018-2487-5 (PMC6303911; doi:10.1186/s12859-018-2487-5)
Supplement: Supplementary file 2 — User guide within the R package FELLA with background, implementation details and three real examples on its usage. (PDF 1096 kb) [file 12859_2018_2487_MOESM2_ESM.pdf]

An R package to enrich metabolomics data: FELLA

Sergio Picart-Armada *[1,2], ***Francesc Fernández-Albert*** [1,2], ***Maria Vinaixa*** [3,4,5], ***Oscar Yanes*** [3,4], ***and Alexandre Perera-Lluna*** [1,2]

[1]B2SLab, Departament d'Enginyeria de Sistemes, Automàtica i Informàtica Industrial, Universitat Politècnica de Catalunya, CIBER-BBN, Barcelona, 08028, Spain
[2]Institut de Recerca Pediàtrica Hospital Sant Joan de Déu, Esplugues de Llobregat, Barcelona, 08950, Spain
[3]Centre for Omic Sciences, Department of Electronic Engineering, Rovira i Virgili University, Reus, 43204, Spain
[4]Metabolomics Platform, Spanish Biomedical Research Centre in Diabetes and Associated Metabolic Disorders, Madrid, 28029, Spain
[5]Manchester Synthetic Biology Research Centre for Fine and Speciality Chemicals, University of Manchester, Manchester, M1 7DN, United Kingdom

*sergi.picart@upc.edu

February 21, 2018

1 Abstract

Pathway enrichment techniques are useful for giving context to experimental metabolomics data. The primary analysis of the raw metabolomics data leads to annotated metabolites with abundance measures. These metabolites are compared between experimental conditions, in order to find discriminative molecular signatures. The secondary analysis of the dataset aims at giving context to the affected metabolites in terms of the prior biological knowledge gathered in metabolic pathways. Several statistical approaches are available to derive a list of prioritised metabolic pathways that relate to the underlying changes in metabolite abundances. However, the interpretation of a prioritised pathway list remains challenging, as pathways are not disjoint and show overlap and cross talk effects. Furthermore, it is not straightforward to automatically propose novel enzymatic targets given a pathway enrichment.

We introduce *FELLA*, an R package to perform a network-based enrichment of a list of affected metabolites. *FELLA* builds a hierarchical network representation of the organism of choice using the Kyoto Encyclopedia of Genes and Genomes, which contains pathways, modules, enzymes, reactions and metabolites. The enrichment is accomplished by applying diffusion algorithms in the knowledge network. Flow is introduced in the metabolites from the input list and propagates to the rest of nodes, resulting in diffusion scores for all the nodes in the network. The top scoring nodes contain not only relevant pathways, but also the intermediate entities that build a plausible explanation on how the input metabolites translate into reported pathways. The highlighted sub-network can shed light on pathway cross talk under the experimental condition and potential enzymatic targets for further study.

The implementation and the programmatic use of *FELLA* is hereby described, along with a graphical user interface that wraps the package functionality. The algorithmic part in *FELLA* was previously validated on the study of an uncharacterised mitochondrial protein. The functionality of *FELLA* has been demonstrated on three public human metabolomics studies, respectively on (a) ovarian cancer cells, (b) dry eye and (c) malaria and other febrile illnesses. *FELLA* has been able to reproduce findings from the original publications and to report sub-network representations that can be manually handled.

2 Introduction

Metabolomics is the science that studies the chemical reactions in living organisms by quantifying their lightweight molecules, called metabolites. The utilities of metabolomics range from disease diagnosis through biomarkers and personalised medicine to the generation of biological knowledge [1].

Metabolomics data is mainly acquired through technologies such as, but not limited to, Nuclear Magnetic Resonance (NMR) and Mass Spectrometry (MS). MS is usually preceded by Liquid Chromatography (LC) or Gas Chromatography (GC) [2]. The primary analysis of the raw metabolomics data can be achieved through publicly available tools: the R packages *xmcs* [3] for peak identification and *CAMERA* [4] for peak annotation. There are pipelines that cover the whole process, for example the online tool MeltDB [5] or the R package *MAIT* [6]. Metabolites found in samples are mapped to specral databases such as the Human Metabolome Database [7].

The secondary analysis, or data interpretation, starts when the metabolites are mapped to a database and their abundances are available [8]. The existence of experimental conditions enables a statistical differential analysis that yields

a set of metabolites that exhibit changes in the intervention. It is, however, increasingly important to understand the underlying biological perturbation by giving context to the affected metabolites rather than focusing on the ability to classify samples through them [1]. Pathway analysis is a fundamental methodology for data interpretation [9] that enriches the affected metabolites with current knowledge on biology, available in pathway databases including the Kyoto Encyclopedia of Genes and Genomes or KEGG [10], Reactome [11] and WikiPathways [12]. Enrichment techniques will be discussed in three categories or generations, according to the classification proposed in the review [9]. Commercial pathway analysis products such as *IPA* (QIAGEN Inc., https://www.qiagenbioinformatics.com/products/ingenuitypathway-analysis) are out of the scope of this work.

The first generation of methods, named over representation analysis (ORA), are based on testing if the proportion of affected metabolites within a pathway is statistically meaningful. ORA is based in statistical tests on probability distribution like the hypergeometric, binomial or chi-squared [9]. ORA is available in tools like the web servers MetaboAnalyst [13] and IMPaLA [14] and the R package *clusterProfiler* [15]. The online resource SubPathwayMiner identifies sub-pathways from KEGG pathways by mining k-cliques in each metabolic pathway prior to ORA. With this strategy, significant sub-regions can be spotted even if the whole pathway is not significant [16].

The second generation of methods, functional class scoring (FCS), uses quantitative data instead and seeks subtle but coordinated changes in the metabolites belonging to a pathway. MSEA [17] in MetaboAnalyst [13] and IMPaLA [14] contain implementations of FCS for metabolomics. The R package *PAPi* calculates pathways activity scores per sample, based on the number of metabolites identified from each pathway and their relative abundances. Significantly affected pathways are found by applying an ANOVA or a t-test on those scores [18]. On the other hand, there is an ensemble approach relying on several pathway-based statistical tests [19] and is available in the R pacakge *EGSEA*.

The third generation, known as pathway topology-based (PT) methods, further includes topological measures of the metabolites in the statistic, accounting for their inequivalence in the metabolic network. PT analyses can be performed using MetaboAnalyst [13], where metabolites are weighted by their centrality within the pathway. The R package *MPINet* builds a pathway-level statistic that accounts for metabolite inequivalence in the global metabolic network and for bias in technical equipment [20].

Another perspective for understanding metabolomics data is through the construction and inquiry of metabolic networks. The *MetScape* plugin [21] within the *Cytoscape* environment [22] is useful for representing metabolite-reaction-enzyme-gene networks. *KEGGGraph* is an R package for constructing metabolic

networks from the KEGG pathways [23]. *MetaboSignal* is an R package for building and examining the topology of gene-metabolite networks [24]. The R package *MetaMapR* helps reduce sparsity in metabolic networks by integrating biochemical transformations, structural similarity, mass spectral similarity and empirical correlation information [25].

Here, we introduce the R package *FELLA* for metabolomics data interpretation that combines concepts from pathway enrichment and network analysis. The main objective of *FELLA* is providing the user with a biological explanation involving biological pathways. *FELLA* starts from a single, comprehensive network consisting of metabolites, reactions, enzymes, modules and pathways as nodes. The list of affected metabolites and the pathways highlighted by *FELLA* are connected through intermediate entities -reactions, enzymes and KEGG modules- and returned as a sub-network. The intermediate entities suggest how the perturbation spreads from metabolites to pathways and how pathways cross talk. The provided enzymes are candidates for further examination, whereas new metabolites might be reported as well. *FELLA* is publicly available in https://github.com/b2slab/FELLA under the GPL-3 license.

3 Methodology

3.1 Implementation details

FELLA is written entirely in R [26] and relies on the *KEGGREST* R package [27] for retreiving KEGG, the *igraph* R package [28] for network analysis and the *shiny* R package [29] for providing a graphical user interface.

FELLA defines two S4 classes for handling its main purposes: a *FELLA.DATA* object that encompasses the knowledge model from KEGG and a *FELLA.USER* object that contains the current analysis by the user. Table 1 contains further details about the slots and sub-slots in each one of these classes, whereas figure 1 depicts the package workflow and main functions.

FELLA contains two vignettes that illustrate its capabilities: (1) a quick-start example with the main functions applied to a toy dataset, and (2) this document, an in-depth demonstration on three real studies. This vignette requires an internet connection and can take up some time and memory to build, as it builds the internal KEGG representation for Homo sapiens on the fly.

Custom class	Slot	Sub-slot	Class	Description
FELLA.DATA	@keggdata	@graph	igraph	Knowledge graph object
		@id2name	list	Dictionary from KEGG ID to common name
		@pvalues.size	matrix	Matrix with largest CC size probabilities
		@id	list	Correspondence between IDs and category
		@status	character	Status indicator of the object
	@hypergeom	@matrix	Matrix	Metabolite-pathway binary relationship
	@diffusion	@matrix	matrix	Matrix to compute diffusion as a matrix-vector product
		@rowSums	vector	Internal data to compute the z-scores
		@squaredRowSums	vector	Internal data to compute the z-scores
	@pagerank	@matrix	matrix	Matrix to compute PageRank as a matrix-vector product
		@rowSums	vector	Internal data to compute the z-scores
		@squaredRowSums	vector	Internal data to compute the z-scores
FELLA.USER	@userinput	@metabolites	vector	KEGG IDs that map to the knowledge graph
		@metabolitesbackground	vector	Background KEGG IDs
		@excluded	vector	Input IDs not mapping to the knowledge graph
	@hypergeom	@valid	logical	Indicator of analysis validity
		@pvalues	vector	Pathway p-values
		@pathhits	vector	Number of hits in each pathway
		@pathbackground	vector	Number of metabolites in each pathway
		@nbackground	numeric	Number of compounds in the background
		@ninput	numeric	Number of compounds in the input
	@diffusion	@valid	logical	Indicator of analysis validity
		@pscores	vector	P-scores for each node in the network
		@approx	character	Chosen approximation
		@niter	numeric	Chosen iterations
	@parerank	@valid	logical	Indicator of analysis validity
		@pscores	vector	P-scores for each node in the network
		@approx	character	Chosen approximation
		@niter	numeric	Chosen iterations

Table 1: **Summary of the S4 classes defined in *FELLA***

3.2 Database and knowledge model

A distinctive feature of *FELLA* is its unique knowledge model. Instead of using individual pathway representations, either as a list of metabolites (ORA) or as a metabolic network (TP), *FELLA* builds a unique network that encompasses all the pathways at once: the KEGG graph. Figure 2 shows the hierarchical representation of the KEGG database, ranging from the small, specific molecular level (metabolite) to the large, complex unit (pathway). Intermediate levels contain, from bottom to top: reactions relating the metabolites, enzymes catalising the reactions and KEGG modules containing the enzymes. More details on the construction and curation of this structure, resemblant to the one used by MetScape [21], can be found in [30]. The enrichment is therefore achieved by finding a sub-network from the whole KEGG graph that is statistically relevant for a list of input metabolites.

As shown in the block (I) of figure 1, the first step is to build a KEGG graph from an organism in KEGG -Homo sapiens by default- using the `buildGraphFromKEGGREST` command. Afterwards, a local database can be built from the KEGG graph through the `buildDataFromGraph` command. The main purposes of `buildDataFromGraph` are to save (1) the matrices that allow computing diffusion and PageRank as a matrix-vector product, and (2) the null distribution of the largest connected component of a k-th order subgraph, with uniformly chosen nodes. Point (1) is required to compute the diffusion scores, whereas (2) is useful for filtering small connected components in the reported subgraphs.

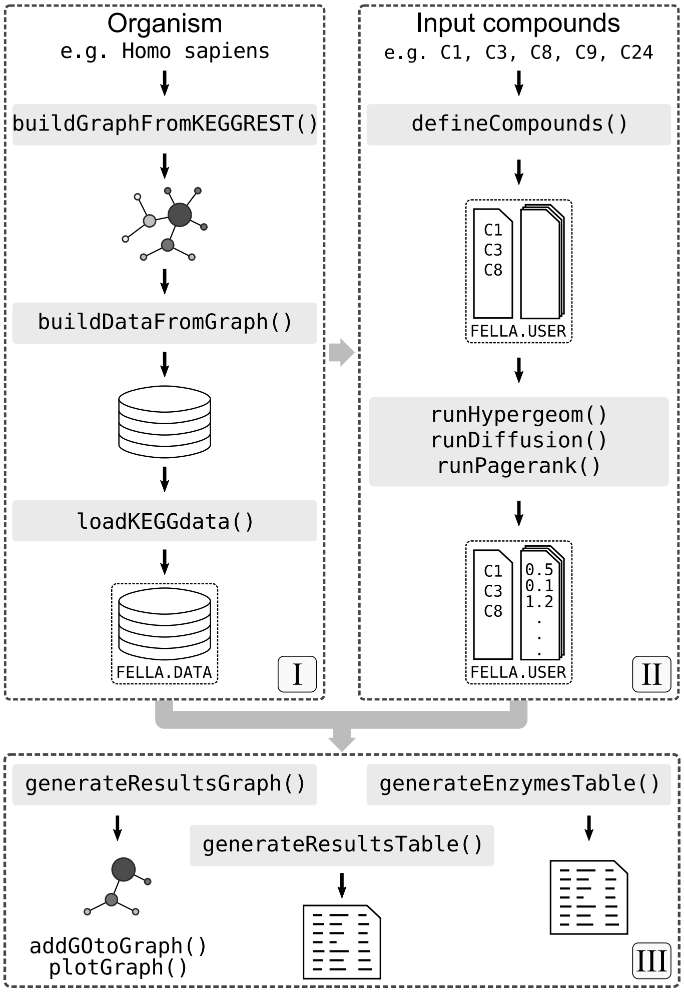

Figure 1: Design of the R package *FELLA*
Block **I** covers the creation of a graph object from an organism code and its database, which can be loaded into a *FELLA.DATA* object. This object is needed in all the following blocks. Block **II** requires block **I** and shows how to map the KEGG identifiers to the database in a *FELLA.USER* object and run the propagation algorithms (diffusion, PageRank) to score all the entities in the graph. Block **III** requires blocks **I** and **II** and exports the results as a sub-network or as a table.

The user should be aware that KEGG is frequently updated and therefore the derived KEGG graph can change between KEGG releases. The metadata from the KEGG version used to build a *FELLA.DATA* object can be retrieved through `getInfo`.

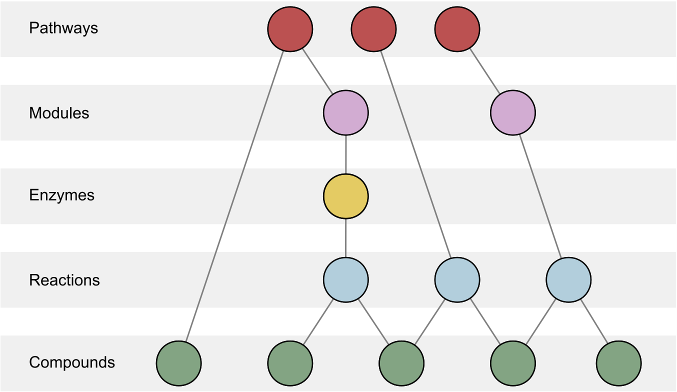

Figure 2: Internal knowledge representation from KEGG
The scheme outlines the KEGG graph, a heterogeneous network whose nodes belong to a category in KEGG: compound, reaction, enzyme, module or pathway. Lower levels are expected to be more specific entities, while top levels are broader concepts. The enrichment procedure starts from input metabolites and extracts a relevant sub-network from the KEGG graph. Figure extractred from [30]

3.3 Enrichment analysis

Once the database is ready as a *FELLA.DATA* object and the input is formatted as a list of KEGG compounds, the enrichment can be performed. The results of the enrichment are stored in a *FELLA.USER* object, possibly using three methodologies described below.

3.3.1 Hypergeometric test

For completeness purposes, the hypergeometric test is included in *FELLA* in the function `runHypergeom`. As in several ORA implementations, the hypergeometric distribution is used to assess whether a biological pathway contains more hits within the input list than expected from chance given its size. Pathways are ranked according to their p-value after multiple testing correction.

Note that the results from this test will differ from a hypergeometric test using the original KEGG pathways, because metabolite-pathway connections are inferred from the KEGG graph. A metabolite is included in a pathway if the pathway can be reached from the metabolite in the upwards-directed KEGG graph, depicted in figure 4. In consequence, metabolites related to the enzymes within a pathway will belong to the pathway, even if they were not in the original definition of the KEGG pathway.

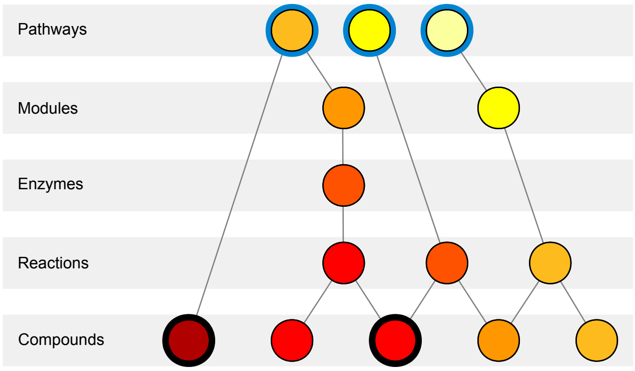

Figure 3: Network setup for the diffusion process
nput metabolites (in black rings) introduce a unitary flow in the network and only the pathway nodes (blue rings) can leak the flow. The final score of the nodes reflects the "temperature" of a stationary state. Figure extractred from [30].

3.3.2 Diffusion

Diffusion algorithms have been extensively used in computational biology. For instance, HotNet is an algorithm for finding sub-networks with a large amount of mutated genes [31], whereas TieDIE attemps to link a source set and a target set of molecular entities through two diffusion processes [32]. Other applications include the prioritisation of disease genes [33] and the prediction of gene function [34].

In *FELLA*, diffusion is a natural way to score all the nodes in the KEGG graph given an input list of metabolites, available using `method = "diffusion"` in the function `runDiffusion`. The input metabolites introduce unitary flow in the network. Flow can only leave the network through pathway nodes, forcing it to propagate through the intermediate entities as well (reactions, enzymes and modules), see figure 3. Further details can found in [30].

However, the diffusion scores are biased due to the network topology [30] and therefore a normalisation step is required. *FELLA* offers a normalisation through a z-score (`approx = "normality"`) or through an empirical p-value (`approx = "simulation"`), both assessing whether the diffusion score of a node is likely to be reached in a permutation analysis, i.e. if the input is random.

The normalisation through the z-scores leads to p-scores, defined as:

$$ps_i = 1 - \Phi(z_i)$$

Where ps_i is the p-score of node i, z_i is its z-score [30] and Φ is the cumulative distribution function of the standard gaussian distribution. Under this definition, nodes are ranked using increasing p-scores.

For completeness, two alternative parametric scores have been added. The heavier-tailed **t**-distribution can be used instead of the gaussian by choosing `approx = "t"` and supplying the desired degrees of freedom ν.

Similarly, the **gamma** distribution can be used through `approx = "gamma"`. The p-score is obtained with

$$ps_i = 1 - F_i(T_i)$$

Being T_i the raw temperature of node i and F_i the cumulative distribution function of a gamma distribution, adjusted by its shape ($\frac{\mu_i^2}{\sigma_i^2}$) and scale ($\frac{\sigma_i^2}{\mu_i}$) parameters. The quantities μ_i and σ_i^2 are the mean and variance of the null temperatures and are analytically known from the null model formulation [30].

3.3.3 PageRank

PageRank [35] offers a scoring method for the nodes in the KEGG graph, based on a random walks approach. The random walks start at the input metabolites and are forced to explore their reachable nodes, see figure 4. As random walks take into account the direction of the edges, PageRank is applied to the upwards-directed KEGG graph (figure 2) in order to force the walks to reach pathway nodes. Nodes that are frequently visited by the random walks earn a higher PageRank, analogously to the diffusion scores. More details about this particular formulation, implemented in `runPagerank`, can be found in [30].

The PageRank scores are statistically normalised, providing the same options as in the diffusion scores in section 3.3.2. Therefore, the argument *approx* can be set to `"simulation"` for the permutation analysis, or to `"normality"`, `"t"` or `"gamma"` for the parametric alternatives.

3.4 Enrichment wrapper

FELLA contains the wrapper `enrich` that maps the KEGG ids and runs the desired enrichment procedure with a single call. This can be convenient for producing compact scripts and running quick analyses.

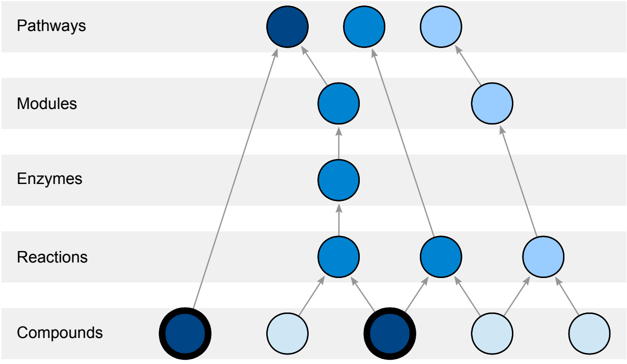

Figure 4: Network setup for PageRank
Input metabolites (in black rings) are the source of random walks that must climb through the graph levels, up to the pathway nodes. Figure extractred from [30].

3.5 Limitations

FELLA currently starts the statistical analysis from a list of affected metabolites. Therefore, it inherits a limitation from ORA methods: the need of choosing a cutoff to derive the list of affected metabolites, assuming that the metabolites stem from a differential abundance analysis.

Another limitation, shared among network-based models, is the incomplete biological knowledge from which the network is built. The knowledge model in *FELLA* might also constraint the complexity of the mechanisms that can be found through it. Processes such as genetic and epigenetic events, or the type and directionality of regulatory events, are not considered at the moment.

The user should be aware that *FELLA* neither builds a dynamic model of the biochemical reactions in the metabolism, nor relies on flux balance analysis. Conversely, *FELLA* is built on a knowledge representation from the biology in KEGG that focuses on offering interpretability to the final user.

4 Case studies

The functionalities of *FELLA* are demonstrated by (1) building a Homo sapiens database and (2) enriching summary metabolomics data from three public datasets.

4.1 Building the database

FELLA requires a database built from KEGG to perform any data enrichment. *FELLA* contains a small example database as a *FELLA.DATA* object, accessible via `data("FELLA.sample")`, but this is a toy example for demonstration purposes, not suited for regular analyses.

Therefore, the database for the corresponding organism has to be built before any analysis is run. The first step is to build the KEGG graph from the current KEGG release with the function `buildGraphFromKEGGREST`. Note that the user can force specific KEGG pathways to be excluded from the graph - the following code removes "overview" metabolic pathways based on KEGG brite.

```
> library(FELLA)
> set.seed(1)
> # Filter overview pathways
> graph <- buildGraphFromKEGGREST(
+     organism = "hsa",
+     filter.path = c("01100", "01200", "01210", "01212", "01230"))
```

Once the KEGG graph is ready, the database will be saved locally using `buildDataFromGraph`. The user can choose which matrices shall be stored using the *matrices* argument - saving both `"diffusion"` and `"pagerank"` might take up to 1GB of disk space.

If the user plans on using the z-score approximation, it is advisable to set the *normality* argument to `c("diffusion", "pagerank")` in order to speed up future computations. Using the z-scores with a custom metabolite background will require the matrices to be saved as well.

Finally, the argument *niter* controls how many random trials are performed in the estimation of the null distribution of the largest connected component of a k-th order random subgraph. As this is a property of the KEGG graph, it is performed once and reused in each analysis. This finds application when filtering small connected components from the reported sub-network, see section 4.2.3.

```
> tmpdir <- paste0(tempdir(), "/my_database")
> # Mke sure the database does not exist from a former vignette build
> # Otherwise the vignette will rise an error
> # because FELLA will not overwrite an existing database
> unlink(tmpdir, recursive = TRUE)
> buildDataFromGraph(
+     keggdata.graph = graph,
+     databaseDir = tmpdir,
```

```
+     internalDir = FALSE,
+     matrices = "diffusion",
+     normality = "diffusion",
+     niter = 50)
```

When the database is available in local, it can be loaded in an *R* session and assigned to a *FELLA.DATA* object using the function `loadKEGGdata`. This should be the only procedure for creating any *FELLA.DATA* object. The user is given the choice of loading the diffusion and pagerank matrices to ease memory saving. In this example, no matrices will be loaded.

```
> fella.data <- loadKEGGdata(
+     databaseDir = tmpdir,
+     internalDir = FALSE,
+     loadMatrix = NULL
+ )
```

The contents of the *FELLA.DATA* object can be summarised as well:

```
> fella.data

General data:
- KEGG graph:
  * Nodes:  9900
  * Edges:  31071
  * Density:  0.0003170507
  * Categories:
    + pathway [315]
    + module [182]
    + enzyme [1110]
    + reaction [4829]
    + compound [3464]
  * Size:  5.6 Mb
- KEGG names are ready.
-----------------------------
Hypergeometric test:
- Matrix not loaded.
-----------------------------
Heat diffusion:
- Matrix not loaded.
- RowSums are ready.
-----------------------------
PageRank:
```

```
- Matrix not loaded.
- RowSums not loaded.
```

The function `getInfo` provides the KEGG release and organism that generated a *FELLA.DATA* object:

```
> cat(getInfo(fella.data))

T01001           Homo sapiens (human) KEGG Genes Database
hsa              Release 85.0+/02-21, Feb 18
                 Kanehisa Laboratories
                 39,556 entries

linked db        pathway
                 brite
                 module
                 ko
                 genome
                 enzyme
                 network
                 disease
                 drug
                 dgroup
                 ncbi-geneid
                 ncbi-proteinid
                 uniprot
```

Please note that the database built for this vignette is stored in a temporary folder and will not be persistent. The user should build his or her own database and save it in a persistent location, either in the package installation directory (`internalDir = TRUE`) or in a custom folder (`internalDir = FALSE`). Internal databases can be listed using `listInternalDatabases`.

A cautionary note if the user is relying on the internal directory: reinstalling *FELLA* will wipe existent databases because its internal directory is overwritten. Also, if the database name already exists when saving a new database, the existing database will be renamed by appending `_old` in order to avoid overwriting.

4.2 Epithelial cells dataset

This example data is extracted from the epithelial cancer cells dataset [36], an in vitro model of dry eye in which the human epithelial cells IOBA-NHC are put under hyperosmotic stress. The original study files are deposited in the Metabolights repository [37] under the identifier MTBLS214: https://www.ebi.ac.uk/metabolights/MTBLS214. The list of metabolites hereby used reflects metabolic changes in "Treatment 1" (24 hours in serum-free media at 380 mOsm) against control (24 hours at 280 mOsm). The metabolites have been extracted from "Table 1" in the original manuscript and mapped to KEGG ids.

4.2.1 Mapping the input metabolites

The input metabolites should be provided as KEGG compound identifiers. If the user starts from another source (common names, HMDB identifiers), tools like the "compound ID conversor" from MetaboAnalyst can be useful for the ID conversion.

```
> compounds.epithelial <- c(
+     "C02862", "C00487", "C00025", "C00064",
+     "C00670", "C00073", "C00588", "C00082", "C00043")
```

The first step is to map the input metabolites to the KEGG graph with `defineCompounds`. This step requires the *FELLA.DATA* object, loaded in section 4.1. The user can impose a custom metabolite background with the *compoundsBackground* argument. By default, all the KEGG compounds in the graph are used.

```
> analysis.epithelial <- defineCompounds(
+     compounds = compounds.epithelial,
+     data = fella.data)
```

Notice that `defineCompounds` throws a warning if any of the input metabolites does not map to the graph. The user can retrieve the mapped and unmapped identifiers through `getInput` and `getExcluded`, respectively.

```
> getInput(analysis.epithelial)

[1] "C00025" "C00043" "C00064" "C00073" "C00082" "C00487" "C00588" "C00670"

> getExcluded(analysis.epithelial)

[1] "C02862"
```

The status of a *FELLA.USER* object can be checked by printing the object.

```
> analysis.epithelial

Compounds in the input: 8
[1] "C00025" "C00043" "C00064" "C00073" "C00082" "C00487" "C00588" "C00670"
Background compounds: all available compounds (default)
-----------------------------
Hypergeometric test: not performed
-----------------------------
Heat diffusion: not performed
-----------------------------
PageRank: not performed
```

4.2.2 Enriching using diffusion

Having mapped the compounds, the enrichment can be performed. In this vignette, only the diffusion method in `runDiffusion` will be applied, although PageRank has an almost identical usage in `runPagerank`.

If the user prefers an explicit permutation analysis, the option `approx = "simulation"` performs the amount of iterations specified in the *niter* argument.

Conversely, if the desired approximation is the z-score (`approx = "normality"`), the process does not require permutations. The z-scores are converted to `p.scores` using the `pnorm` routine. Likewise, `approx = "t"` and `approx = "gamma"` respectively rely on `pt` and `pgamma`. Section 3.3.2 contains further details on the scores.

This example applies `approx = "normality"`, a fast option. For a comparison between prioritisations using Monte Carlo trials or the parametric z-score, the user can is referred to [30].

```
> analysis.epithelial <- runDiffusion(
+     object = analysis.epithelial,
+     data = fella.data,
+     approx = "normality")
```

The *FELLA.USER* object has been updated with the `p.scores` from the diffusion results:

```
> analysis.epithelial

Compounds in the input: 8
```

```
[1] "C00025" "C00043" "C00064" "C00073" "C00082" "C00487" "C00588" "C00670"
Background compounds: all available compounds (default)
-----------------------------
Hypergeometric test: not performed
-----------------------------
Heat diffusion: ready.
P-scores under 0.05:  261
-----------------------------
PageRank: not performed
```

At this point, the subgraph consisting of top scoring nodes can be plotted in a heterogeneous network layout. In the presence of signal, this subgraph will exhibit large connected components and contain nodes from all the levels in the KEGG graph. It is also expected that the algorithm gives a high priority to the metabolites specified in the input, although not all of them must necessarily be top ranked.

Therefore, the user should expect to find the presence of intermediate entities (reactions, enzymes and modules) that connect the input to relevant KEGG pathways. Note that *FELLA* can also pinpoint new KEGG compounds as potentially relevant.

In this example, the plot is limited to 150 nodes using the *nlimit* argument from `plot`.

```
> nlimit <- 150
> vertex.label.cex <- .5
> plot(
+     analysis.epithelial,
+     method = "diffusion",
+     data = fella.data,
+     nlimit = nlimit,
+     vertex.label.cex = vertex.label.cex)
```

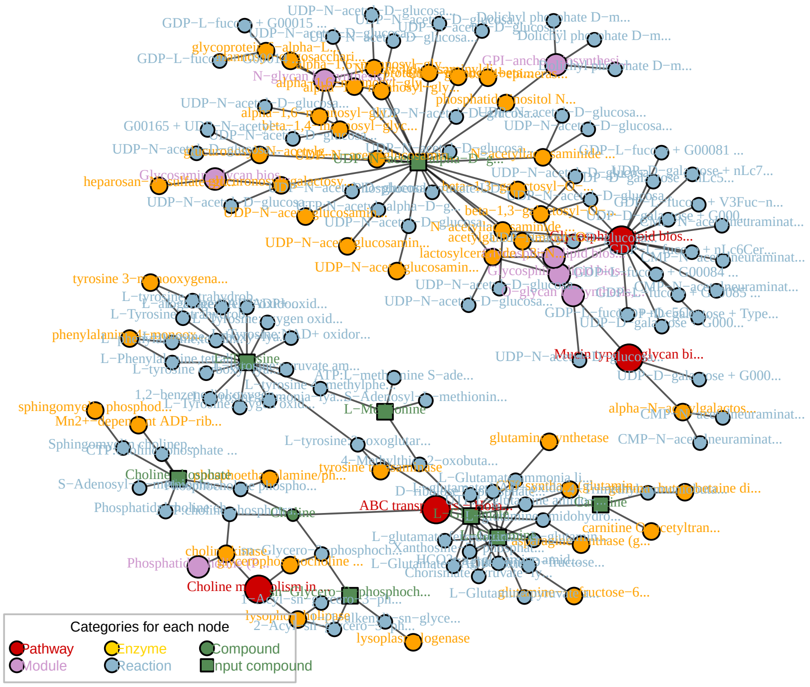

In the original work [36], the activation of the **glycerophosphocholine synthesis** rather than the **carnitine** response is a main result. *FELLA* highlights[1] the related pathway *choline metabolism in cancer* and the *choline* metabolite as well. Another key process is the **O-linked glycosilation**, which is close to the KEGG module *O-glycan biosynthesis, mucin type core* and to the KEGG pathway *Mucin type O-glycan biosynthesis*. Finally, *FELLA* reproduces the finding of **UAP1** by reporting the enzyme *2.7.7.23*, named *UDP-N-acetylglucosamine diphosphorylase*. **UAP1** is a key protein in the study, pinpointed by iTRAQ and validated via western blot.

[1]This analysis is subject to KEGG release 83.0, from August 17th, 2017. Posterior KEGG releases might alter the reported sub-network

4.2.3 Exporting the results

After an initial exploration of the results, these can be exported using three functions that lead to network and tabular formats.

The top scoring nodes can be exported as a network in *igraph* with the function `generateResultsGraph`. The number k of nodes in the subgraph is controlled by the most stringent filter between *nlimit* (limit on the number of nodes) and *threshold* (limit on the `p.score`).

Once k is determined, the argument *thresholdConnectedComponent* further filters small connected components from the subgraph, implying that the resulting subgraph can have less than k nodes. A connected component of order r will be kept only if the probability that a random subgraph of order k contains a connected component of order at least r is smaller than the specified threshold. In other words, small connected components can arise from random sampling of the subgraph, whereas larger connected components are highly unlikely under a uniform sampling. The user can filter connected components that are too small to be meaningful in that sense.

Lastly, the argument *LabelLengthAtPlot* allows to truncate the KEGG names at the given number of characters for visualisation purposes.

```
> g <- generateResultsGraph(
+     object = analysis.epithelial,
+     method = "diffusion",
+     nlimit = nlimit,
+     data = fella.data)
> g

IGRAPH 305e4ef UNW- 150 180 --
+ attr: organism (g/c), name (v/c), com (v/n), NAME (v/x), entrez
| (v/x), label (v/c), input (v/l), weight (e/n)
+ edges from 305e4ef (vertex names):
 [1] hsa00512--M00056    hsa00601--M00070    hsa00601--M00071
 [4] M00056  --2.4.1.102 M00075  --2.4.1.143 M00075  --2.4.1.144
 [7] M00075  --2.4.1.145 hsa00601--2.4.1.146 M00056  --2.4.1.147
[10] hsa00601--2.4.1.149 hsa00601--2.4.1.150 M00075  --2.4.1.155
[13] M00065  --2.4.1.198 M00075  --2.4.1.201 M00070  --2.4.1.206
[16] M00071  --2.4.1.206 M00059  --2.4.1.223 M00059  --2.4.1.224
[19] M00075  --2.4.1.68  hsa00512--2.4.99.3  hsa05231--2.7.1.32
+ ... omitted several edges
```

The exported (sub)graph can be further complemented with data from GO, the Gene Ontology [38]. Specifically, the enzymes can be equipped with annotations from their underlying genes in any ontology from GO. Note that this requires additional packages: *biomaRt* and *org.Hs.eg.db*. The latter should be changed in case the analysis and the database are not from Homo sapiens.

The function `addGOToGraph` achieves this by accepting a query GO term and computing the semantic similarity of all the genes within each enzyme to the query GO term. The semantic similarity is detailed and implemented in the package *GOSemSim* [39].

In the current example, enzymes are going to be compared to the GO cellular component term mitochondrion. Enzymes that contain genes whose cellular component is closer or coincident with the mitochondrion will be highlighted.

```
> # GO:0005739 is the term for mitochondrion
> g.go <- addGOToGraph(
+     graph = g,
+     GOterm = "GO:0005739",
+     godata.options = list(
+         OrgDb = "org.Hs.eg.db", ont = "CC"),
+     mart.options = list(
+         biomart = "ensembl", dataset = "hsapiens_gene_ensembl"))
> g.go

IGRAPH 305e4ef UNW- 150 180 --
+ attr: organism (g/c), name (v/c), com (v/n), NAME (v/x), entrez
| (v/x), label (v/c), input (v/l), GO (v/x), GO.simil (v/x), weight
| (e/n)
+ edges from 305e4ef (vertex names):
 [1] hsa00512--M00056    hsa00601--M00070    hsa00601--M00071
 [4] M00056  --2.4.1.102 M00075  --2.4.1.143 M00075  --2.4.1.144
 [7] M00075  --2.4.1.145 hsa00601--2.4.1.146 M00056  --2.4.1.147
[10] hsa00601--2.4.1.149 hsa00601--2.4.1.150 M00075  --2.4.1.155
[13] M00065  --2.4.1.198 M00075  --2.4.1.201 M00070  --2.4.1.206
[16] M00071  --2.4.1.206 M00059  --2.4.1.223 M00059  --2.4.1.224
+ ... omitted several edges
```

Plotting the graph with the function `plotGraph` reveals the addition of the GO term due to a slight change in the plotting legend. Enzyme nodes have a different shape and their colour scale reflects their degree of similarity to the queried GO term.

```
> plotGraph(
+     g.go,
+     vertex.label.cex = vertex.label.cex)
```

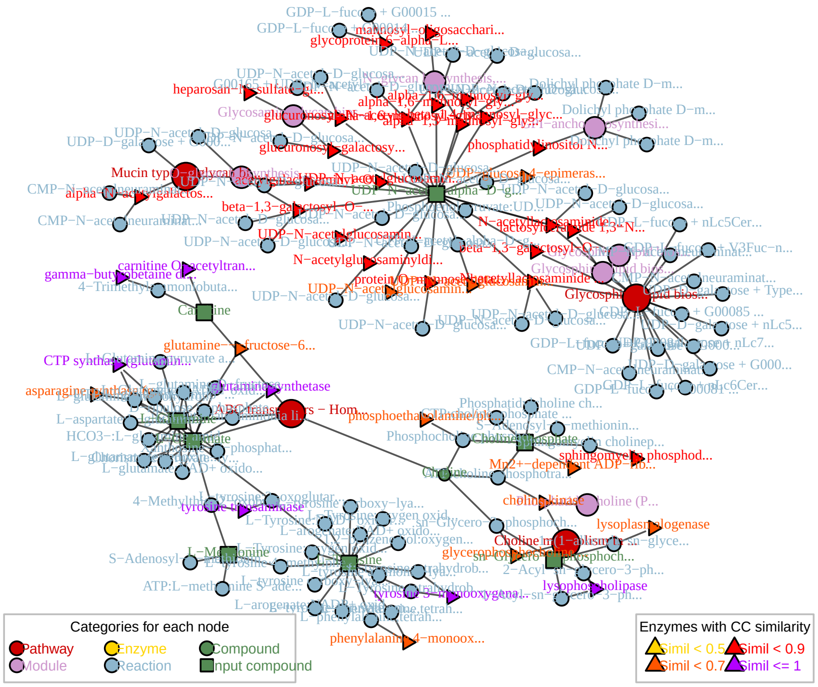

The second way to export the enrichment results is to write the data from the KEGG entries in the top k p.scores using `generateResultsTable`. This function accepts arguments similar to those in `generateResultsTable`.

```
> tab.all <- generateResultsTable(
+     method = "diffusion",
+     nlimit = 100,
+     object = analysis.epithelial,
+     data = fella.data)
> # Show head of the table
> knitr::kable(head(tab.all), format = "latex")
```

KEGG.id	Entry.type	KEGG.name	p.score
hsa00512	pathway	Mucin type O-glycan biosynthesis - Homo sapie...	1.32e-05
hsa05231	pathway	Choline metabolism in cancer - Homo sapiens (...	1.00e-06
M00056	module	O-glycan biosynthesis, mucin type core	1.20e-06
M00059	module	Glycosaminoglycan biosynthesis, heparan sulfa...	1.00e-06
M00075	module	N-glycan biosynthesis, complex type	1.00e-06
1.14.11.1	enzyme	gamma-butyrobetaine dioxygenase	1.00e-06

The last exporting option, `generateEnzymesTable`, is to a tabular format with details from the enzymes reported among the top k KEGG entries. In particular, the table contains the genes that belong to each enzyme family, separated by semicolons.

```
> tab.enzyme <- generateEnzymesTable(
+     method = "diffusion",
+     nlimit = 100,
+     object = analysis.epithelial,
+     data = fella.data)
> # Show head of the table
> knitr::kable(head(tab.enzyme, 10), format = "latex")
```

EC_number	p.score	EC_name	Genes
2.3.1.7	1e-06	carnitine O-acetyltransferase	1384
1.14.11.1	1e-06	gamma-butyrobetaine dioxygenase	8424
3.1.3.75	1e-06	phosphoethanolamine/phosphocholine phosphatas...	162466
3.1.4.2	1e-06	glycerophosphocholine phosphodiesterase	56261
3.6.1.53	1e-06	Mn2+-dependent ADP-ribose/CDP-alcohol diphosp...	56985
2.7.8.17	1e-06	UDP-N-acetylglucosamine—lysosomal-enzyme N-...	79158
3.1.4.12	1e-06	sphingomyelin phosphodiesterase	339221;55512;55627;660
2.4.1.143	1e-06	alpha-1,6-mannosyl-glycoprotein 2-beta-N-acet...	4247
2.4.1.144	1e-06	beta-1,4-mannosyl-glycoprotein 4-beta-N-acety...	4248
2.4.1.145	1e-06	alpha-1,3-mannosyl-glycoprotein 4-beta-N-acet...	11282;11320;152586;258

The three exporting options shown above are included in the wrapper function `exportResults`, using `format = "csv"` for the general tabular data, `format = "enzyme"` for the enzyme tabular data and `format = "igraph"` for saving an `.RData` object with the igraph sub-network object.

For instance, the general tabular data:

```
> tmpfile <- tempfile()
> exportResults(
```

```
+     format = "csv",
+     file = tmpfile,
+     method = "diffusion",
+     object = analysis.epithelial,
+     data = fella.data)
```

If the argument *format* is none of the former, *FELLA* saves the sub-network using `write.graph` from the *igraph* package with the desired format.

```
> tmpfile <- tempfile()
> exportResults(
+     format = "pajek",
+     file = tmpfile,
+     method = "diffusion",
+     object = analysis.epithelial,
+     data = fella.data)
```

4.2.4 Deploying the graphical user interface

FELLA is equipped with a graphical user interface that eases data analysis without learning the package syntax. The app is divided in the following tabs:

- Compounds upload (figure 5): contains a general description of the tabs and a handle to submit the input metabolite list as a text file. Examples are provided as well. The right panel shows the mapped and the mismatching compounds with regard to the default database.
- Advanced options (figure 6): widgets that contain the main function arguments for customising the enrichment procedure. Allows database choice from the internal package directory, method and approximation choice and parameter tweaking. It also allows defining a GO label for the semantic similarity analysis on the reported enzymes.
- Results (figure 7): interactive plot with the sub-graph with the top k KEGG entries. Nodes can be selected, queried and link to the KEGG entries when hovered. Below the network lies an interactive table with the graph nodes, allowing the user to look into particular entries.
- Export (figure 8): several tabular and network exporting options.

The app is based on *shiny* [29] and can be launched through `launchApp`.

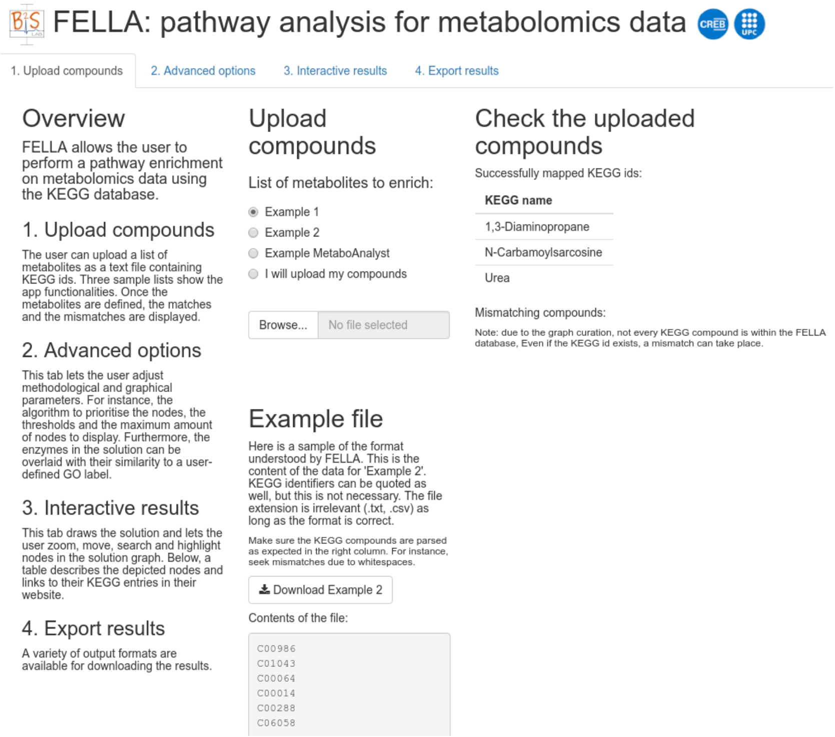

Figure 5: **Graphical interface: compounds upload**

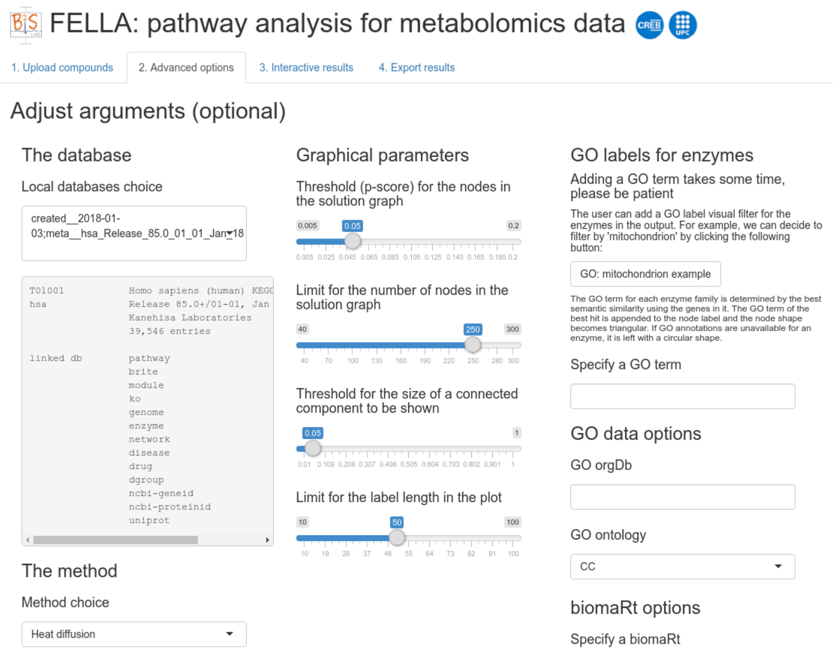

Figure 6: **Graphical interface: advanced options**

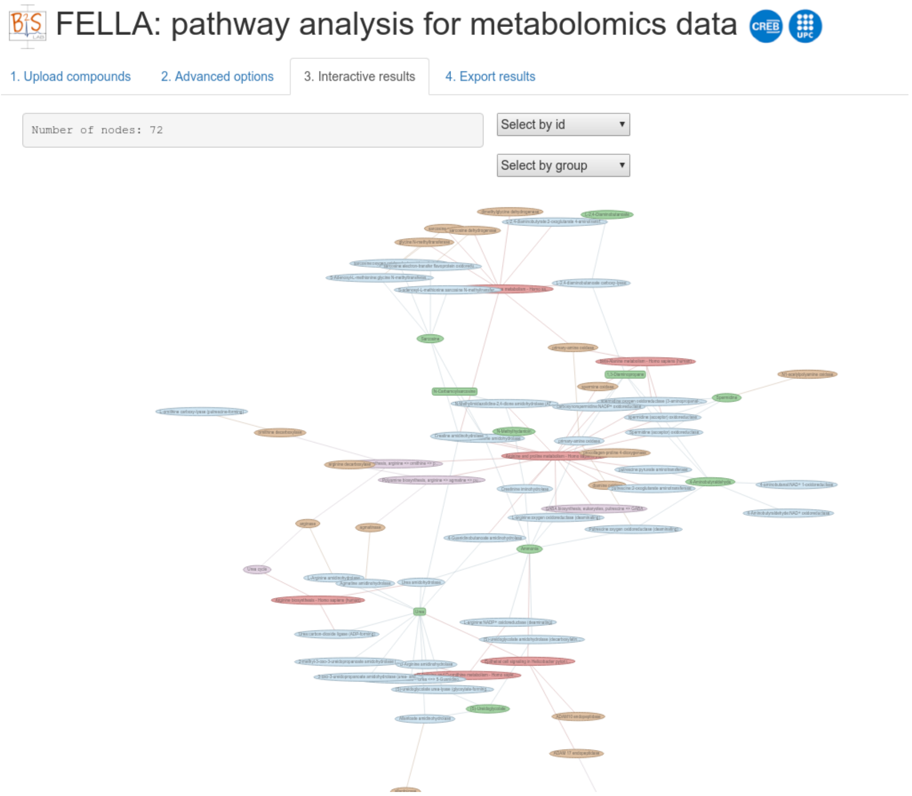

Figure 7: Graphical interface: results

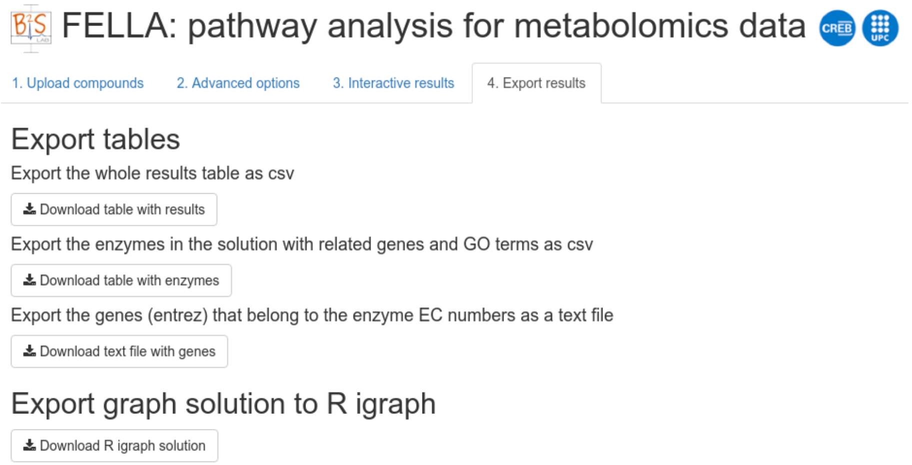

Figure 8: Graphical interface: export

4.2.5 Helper functions

FELLA is equipped with helper functions that ease the user experience and avoid direct manipulation of the S4 classes. Some of them have been already introduced - a complete enumeration of the exported functions is hereby provided.

Functions of the type `get-` ease object and slot retrieval, with the following possibilities: `getBackground`, `getExcluded`, `getInfo`, `getInput`, `getName`, `getPscores`.

On the other hand, functions starting by `list-` provide general purpose data about the package (`listMethods`, `listApprox`, `listCategories`) and a listing of the available internal databases (`listInternalDatabases`).

Finally, functions starting by `is-` check if an object belongs to a certain class: `is.FELLA.DATA` and `is.FELLA.USER`.

4.3 Ovarian cancer cells dataset

The next example has been extracted from the study on metabolic responses of ovarian cancer cells [40]. The original files can be found in the MTBLS150 study in the Metabolights respository: https://www.ebi.ac.uk/metabolights/MTBLS150. OCSCs are isogenic ovarian cancer stem cells derived from the OVCAR-3 ovarian cancer cells. The abundances of six metabolites are affected by the exposure to several environmental conditions: glucose deprivation, hypoxia and ischemia (column "All" in "Figure 3" from their main manuscript).

The common names have been converted to KEGG ids prior to applying *FELLA*. The analysis is performed using the wrapper `enrich` that maps the compounds to the internal representation and runs the desired methods.

```
> compounds.ovarian <- c(
+     "C00275", "C00158", "C00042",
+     "C00346", "C00122", "C06468")
> analysis.ovarian <- enrich(
+     compounds = compounds.ovarian,
+     data = fella.data,
+     methods = "diffusion")
> nlimit <- 150
> plot(
+     analysis.ovarian,
+     method = "diffusion",
+     data = fella.data,
+     nlimit = nlimit,
+     vertex.label.cex = vertex.label.cex,
+     plotLegend = FALSE)
```

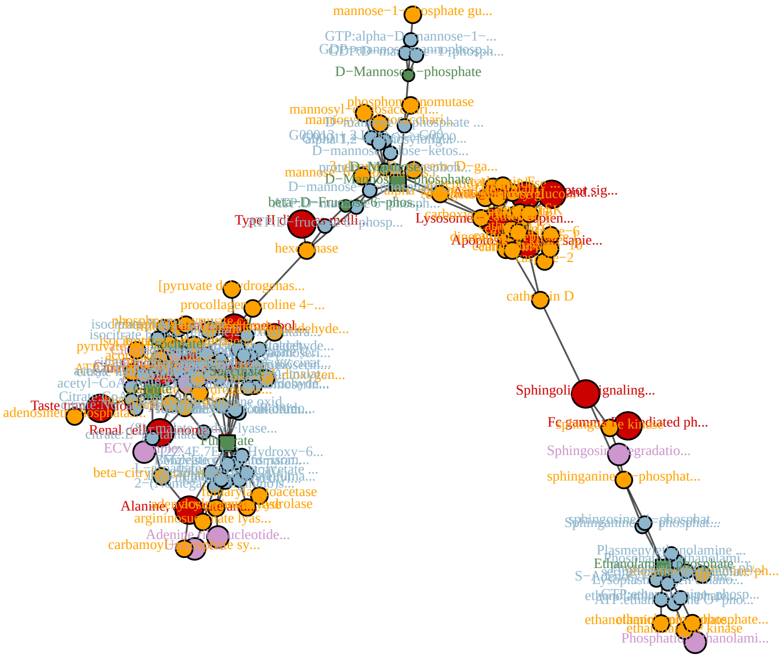

The resulting subnetwork[2] reports several **TCA cycle**-related entities, also reported by the authors and by previous work [41]. It also mentions *sphingosine degradation*, closely related to the reported **sphingosine metabolism** in the original work. Enzymes that have been formerly related to cancer are suggested within the TCA cycle, like *fumarate hydratase* [42, 43, 41] *succinate dehydrogenase* [44, 41] and *aconitase* [45]. Another suggestion is *lysosome* - lysosomes suffer changes in cancer cells and directly affect apoptosis [46]. Finally, the graph contains several *hexokinases*, potential targets to disrupt glycolysis, a fundamental need in cancer cells [47].

[2] This analysis is subject to KEGG release 83.0, from August 17th, 2017. Posterior KEGG releases might alter the reported subnetwork

4.4 Malaria dataset

The metabolites in the last example are related to the distinction between malaria and other febrile ilnesses in [48]. The study files can be found under the MTBLS315 identifier in Metabolights: https://www.ebi.ac.uk/metabolights/MTBLS315. Specifically, the list of KEGG identifiers has been extracted from the supplementary data spreadsheet, using all the possible KEGG matches for the "non malaria" patient group.

```
> compounds.malaria <- c(
+     "C05471", "C14831", "C02686", "C06462", "C00735", "C14833",
+     "C18175", "C00550", "C01124", "C05474", "C05469")
> analysis.malaria <- enrich(
+     compounds = compounds.malaria,
+     data = fella.data,
+     methods = "diffusion")
> nlimit <- 50
> plot(
+     analysis.malaria,
+     method = "diffusion",
+     data = fella.data,
+     nlimit = nlimit,
+     vertex.label.cex = vertex.label.cex,
+     plotLegend = FALSE)
```

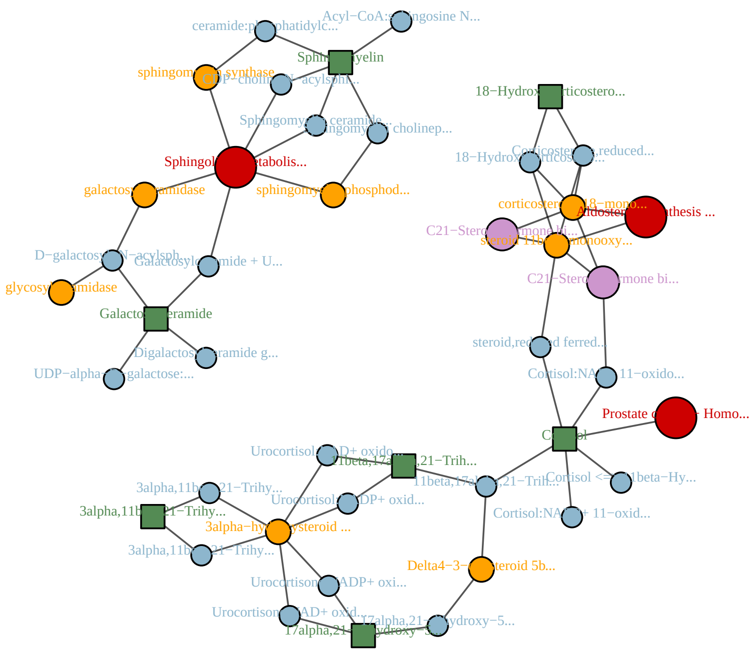

In this case, the depicted subnetwork[3] contains the modules *C21-Steroid hormone biosynthesis, progesterone => corticosterone/aldosterone* and *C21-Steroid hormone biosynthesis, progesterone => cortisol/cortisone*, related to the **corticosteroids** as a main pathway reported in the original text. This is part of the also reported *Aldosterone synthesis and secretion*; aldosterone is known to show changes related to fever as a metabolic response to infection [49]. Another plausible hit in the sub-network is *linoleic acid metabolism*, as erythrocytes infected by various malaria parasytes can be enriched in linoleic acid [50]. In addition, the pathway *sphingolipid metabolism* can play a role in the immune response [51, 52]. As for the enzymes, *3alpha-hydroxysteroid 3-dehydrogenase (Si-specific)* and *Delta4-3-oxosteroid 5beta-reductase* are related to three input metabolites each and might be candidates for further examination.

[3] This analysis is subject to KEGG release 83.0, from August 17th, 2017. Posterior KEGG releases might alter the reported sub-network

5 Conclusions

The *FELLA* R package provides a simple, programmatic and intuitive enrichment tool for metabolomics summary data. Starting from a list of metabolites, *FELLA* not only pinpoints relevant pathways but also intermediate reactions, enzymes and modules that links the input metabolites to the pathways. The reported entries have a network structure focused on interpretability and new hypotheses generation, giving a richer perspective than classical pathway enrichment tools. This comprehensive layout can also suggest potential enzymes and new metabolites for further study. Finally, *FELLA* comes equipped with a graphical user interface that promotes its usage to a wider audience and offers interactive sub-network examination.

6 Funding

This work was supported by the Spanish Ministry of Economy and Competitiveness (MINECO) [BFU2014-57466-P to O.Y., TEC2014-60337-R and DPI2017-89827-R to A.P.]. O.Y., A.P. and S.P. thank for funding the Spanish Biomedical Research Centre in Diabetes and Associated Metabolic Disorders (CIBERDEM) and the Networking Biomedical Research Centre in the subject area of Bioengineering, Biomaterials and Nanomedicine (CIBER-BBN), both initiatives of Instituto de Investigación Carlos III (ISCIII). SP. thanks the AGAUR FI-scholarship programme.

References

[1] Rasmus Madsen, Torbjörn Lundstedt, and Johan Trygg. Chemometrics in metabolomics – a review in human disease diagnosis. *Analytica chimica acta*, 659(1):23–33, 2010.

[2] Wolfram Weckwerth. Annual Review of Plant Biology. 54(1):669–689, 2003.

[3] Colin A Smith, Elizabeth J Want, Grace O'Maille, Ruben Abagyan, and Gary Siuzdak. Xcms: processing mass spectrometry data for metabolite profiling using nonlinear peak alignment, matching, and identification. *Analytical chemistry*, 78(3):779–787, 2006.

[4] Carsten Kuhl, Ralf Tautenhahn, Christoph Bottcher, Tony R Larson, and Steffen Neumann. Camera: an integrated strategy for compound spectra extraction and annotation of liquid chromatography/mass spectrometry data sets. *Analytical chemistry*, 84(1):283–289, 2011.

[5] Nikolas Kessler, Heiko Neuweger, Anja Bonte, Georg Langenkämper, Karsten Niehaus, Tim W Nattkemper, and Alexander Goesmann. Meltdb 2.0–advances of the metabolomics software system. *Bioinformatics*, 29(19):2452–2459, 2013.

[6] Francesc Fernández-Albert, Rafael Llorach, Cristina Andrés-Lacueva, and Alexandre Perera. An R package to analyse LC/MS metabolomic data: MAIT (Metabolite Automatic Identification Toolkit). *Bioinformatics*, 30(13):1937–1939, 2014.

[7] David S Wishart, Timothy Jewison, An Chi Guo, Michael Wilson, Craig Knox, Yifeng Liu, Yannick Djoumbou, Rupasri Mandal, Farid Aziat, Edison Dong, et al. Hmdb 3.0 – the human metabolome database in 2013. *Nucleic acids research*, 41(D1):D801–D807, 2012.

[8] Monica Chagoyen and Florencio Pazos. Tools for the functional interpretation of metabolomic experiments. *Briefings in bioinformatics*, 14(6):737–744, 2012.

[9] Purvesh Khatri, Marina Sirota, and Atul J. Butte. Ten Years of Pathway Analysis: Current Approaches and Outstanding Challenges. *PLoS Computational Biology*, 8(2), 2012.

[10] Minoru Kanehisa, Susumu Goto, Yoko Sato, Miho Furumichi, and Mao Tanabe. Kegg for integration and interpretation of large-scale molecular data sets. *Nucleic acids research*, 40(D1):D109–D114, 2011.

[11] Antonio Fabregat, Konstantinos Sidiropoulos, Phani Garapati, Marc Gillespie, Kerstin Hausmann, Robin Haw, Bijay Jassal, Steven Jupe, Florian Korninger, Sheldon McKay, et al. The reactome pathway knowledgebase. *Nucleic acids research*, 44(D1):D481–D487, 2015.

[12] Martina Kutmon, Anders Riutta, Nuno Nunes, Kristina Hanspers, Egon L Willighagen, Anwesha Bohler, Jonathan Mélius, Andra Waagmeester, Sravanthi R Sinha, Ryan Miller, et al. Wikipathways: capturing the full diversity of pathway knowledge. *Nucleic acids research*, 44(D1):D488–D494, 2015.

[13] Jianguo Xia, Igor V Sinelnikov, Beomsoo Han, and David S Wishart. MetaboAnalyst 3.0 – making metabolomics more meaningful. *Nucleic Acids Research*, 43(Web Server issue):W251–W257, 2015.

[14] Atanas Kamburov, Rachel Cavill, Timothy MD Ebbels, Ralf Herwig, and Hector C Keun. Integrated pathway-level analysis of transcriptomics and metabolomics data with impala. *Bioinformatics*, 27(20):2917–2918, 2011.

[15] Guangchuang Yu, Li-Gen Wang, Yanyan Han, and Qing-Yu He. clusterprofiler: an r package for comparing biological themes among gene clusters. *Omics: a journal of integrative biology*, 16(5):284–287, 2012.

[16] Chunquan Li, Xia Li, Yingbo Miao, Qianghu Wang, Wei Jiang, Chun Xu, Jing Li, Junwei Han, Fan Zhang, Binsheng Gong, et al. Subpathwayminer: a software package for flexible identification of pathways. *Nucleic acids research*, 37(19):e131–e131, 2009.

[17] Jianguo Xia and David S Wishart. Msea: a web-based tool to identify biologically meaningful patterns in quantitative metabolomic data. *Nucleic acids research*, 38(suppl_2):W71–W77, 2010.

[18] Raphael BM Aggio, Katya Ruggiero, and Silas Granato Villas-Bôas. Pathway activity profiling (papi): from the metabolite profile to the metabolic pathway activity. *Bioinformatics*, 26(23):2969–2976, 2010.

[19] Monther Alhamdoosh, Milica Ng, Nicholas J Wilson, Julie M Sheridan, Huy Huynh, Michael J Wilson, and Matthew E Ritchie. Combining multiple tools outperforms individual methods in gene set enrichment analyses. *Bioinformatics*, 33(3):414–424, 2017.

[20] Feng Li, Yanjun Xu, Desi Shang, Haixiu Yang, Wei Liu, Junwei Han, Zeguo Sun, Qianlan Yao, Chunlong Zhang, Jiquan Ma, et al. Mpinet: Metabolite pathway identification via coupling of global metabolite network structure and metabolomic profile. *BioMed research international*, 2014, 2014.

[21] Alla Karnovsky, Terry Weymouth, Tim Hull, V Glenn Tarcea, Giovanni Scardoni, Carlo Laudanna, Maureen A Sartor, Kathleen A Stringer, HV Jagadish, Charles Burant, et al. Metscape 2 bioinformatics tool for the analysis and visualization of metabolomics and gene expression data. *Bioinformatics*, 28(3):373–380, 2011.

[22] Michael E Smoot, Keiichiro Ono, Johannes Ruscheinski, Peng-Liang Wang, and Trey Ideker. Cytoscape 2.8: new features for data integration and network visualization. *Bioinformatics*, 27(3):431–432, 2010.

[23] Jitao David Zhang and Stefan Wiemann. Kegggraph: a graph approach to kegg pathway in r and bioconductor. *Bioinformatics*, 25(11):1470–1471, 2009.

[24] Andrea Rodriguez-Martinez, Rafael Ayala, Joram M Posma, Ana L Neves, Dominique Gauguier, Jeremy K Nicholson, and Marc-Emmanuel Dumas. Metabosignal: a network-based approach for topological analysis of metabotype regulation via metabolic and signaling pathways. *Bioinformatics*, 33(5):773–775, 2017.

[25] Dmitry Grapov, Kwanjeera Wanichthanarak, and Oliver Fiehn. Metamapr: pathway independent metabolomic network analysis incorporating unknowns. *Bioinformatics*, 31(16):2757–2760, 2015.

[26] R Core Team. *R: A Language and Environment for Statistical Computing*. R Foundation for Statistical Computing, Vienna, Austria, 2017. URL: https://www.R-project.org/.

[27] Dan Tenenbaum. *KEGGREST: Client-side REST access to KEGG*, 2017. R package version 1.16.1.

[28] Gabor Csardi and Tamas Nepusz. The igraph software package for complex network research. *InterJournal*, Complex Systems:1695, 2006.

[29] Winston Chang, Joe Cheng, JJ Allaire, Yihui Xie, and Jonathan McPherson. *shiny: Web Application Framework for R*, 2017. R package version 1.0.5. URL: https://CRAN.R-project.org/package=shiny.

[30] Sergio Picart-Armada, Francesc Fernández-Albert, Maria Vinaixa, Miguel Angel Rodriguez, Suvi Aivio, Travis H. Stracker, Oscar Yanes, and Alexandre Perera-Lluna. Null diffusion-based enrichment for metabolomics data. *PLOS ONE*, 12(12):e0189012, 2017.

[31] Fabio Vandin, Eli Upfal, and Benjamin J Raphael. Algorithms for detecting significantly mutated pathways in cancer. *J. Comput. Biol.*, 18(3):507–522, 2011.

[32] Evan O Paull, Daniel E Carlin, Mario Niepel, Peter K Sorger, David Haussler, and Joshua M Stuart. Discovering causal pathways linking genomic events to transcriptional states using Tied Diffusion Through Interacting Events (TieDIE). *Bioinformatics*, 29(21):2757–2764, 2013.

[33] Insuk Lee, U Martin Blom, Peggy I Wang, Jung Eun Shim, and Edward M Marcotte. Prioritizing candidate disease genes by network-based boosting of genome-wide association data. *Genome research*, 21(7):1109–1121, 2011.

[34] Sara Mostafavi, Debajyoti Ray, David Warde-Farley, Chris Grouios, and Quaid Morris. Genemania: a real-time multiple association network integration algorithm for predicting gene function. *Genome biology*, 9(1):S4, 2008.

[35] Lawrence Page, Sergey Brin, Rajeev Motwani, and Terry Winograd. The pagerank citation ranking: Bringing order to the web. Technical report, Stanford InfoLab, 1999.

[36] Liyan Chen, Jing Li, Tiannan Guo, Sujoy Ghosh, Siew Kwan Koh, Dechao Tian, Liang Zhang, Deyong Jia, Roger W Beuerman, Ruedi Aebersold, et al. Global metabonomic and proteomic analysis of human conjunctival epithelial cells (IOBA-NHC) in response to hyperosmotic stress. *Journal of proteome research*, 14(9):3982–3995, 2015.

[37] Kenneth Haug, Reza M Salek, Pablo Conesa, Janna Hastings, Paula de Matos, Mark Rijnbeek, Tejasvi Mahendraker, Mark Williams, Steffen Neumann, Philippe Rocca-Serra, et al. MetaboLights – an open-access general-purpose repository for metabolomics studies and associated meta-data. *Nucleic Acids Res.*, 41(D1):D781–D786, 2012.

[38] Gene Ontology Consortium et al. Gene ontology consortium: going forward. *Nucleic acids research*, 43(D1):D1049–D1056, 2015.

[39] Guangchuang Yu, Fei Li, Yide Qin, Xiaochen Bo, Yibo Wu, and Shengqi Wang. Gosemsim: an r package for measuring semantic similarity among go terms and gene products. *Bioinformatics*, 26(7):976–978, 2010.

[40] Kathleen A Vermeersch, Lijuan Wang, John F McDonald, and Mark P Styczynski. Distinct metabolic responses of an ovarian cancer stem cell line. *BMC systems biology*, 8(1):134, 2014.

[41] Patrick Pollard, Noel Wortham, and Ian Tomlinson. The TCA cycle and tumorigenesis: the examples of fumarate hydratase and succinate dehydrogenase. *Annals of Medicine*, 35(8):634–639, 2003. doi:10.1080/07853890310018458.

[42] M Pithukpakorn, M-H Wei, O Toure, P J Steinbach, G M Glenn, B Zbar, W M Linehan, and J R Toro. Fumarate hydratase enzyme activity in lymphoblastoid cells and fibroblasts of individuals in families with hereditary leiomyomatosis and renal cell cancer. *Journal of medical genetics*, 43(9):755–62, 2006. doi:10.1136/jmg.2006.041087.

[43] Heli J. Lehtonen, Ignacio Blanco, Jose M. Piulats, Riitta Herva, Virpi Launonen, and Lauri A. Aaltonen. Conventional renal cancer in a patient with fumarate hydratase mutation. *Human Pathology*, 38(5):793–796, 2007. doi:10.1016/j.humpath.2006.10.011.

[44] Ying Ni, Kevin M. Zbuk, Tammy Sadler, Attila Patocs, Glenn Lobo, Emily Edelman, Petra Platzer, Mohammed S. Orloff, Kristin A. Waite, and Charis Eng. Germline Mutations and Variants in the Succinate Dehydrogenase Genes in Cowden and Cowden-like Syndromes. *American Journal of Human Genetics*, 83(2):261–268, 2008. doi:10.1016/j.ajhg.2008.07.011.

[45] Keshav K Singh, Mohamed M Desouki, Renty B Franklin, and Leslie C Costello. Mitochondrial aconitase and citrate metabolism in malignant and nonmalignant human prostate tissues. *Molecular cancer*, 5:14, 2006. doi:10.1186/1476-4598-5-14.

[46] Thomas Kirkegaard and Marja Jäättelä. Lysosomal involvement in cell death and cancer. *Biochimica et Biophysica Acta - Molecular Cell Research*, 1793(4):746–754, 2009. doi:10.1016/j.bbamcr.2008.09.008.

[47] William G Kaelin and Craig B Thompson. Q&A: Cancer: clues from cell metabolism. *Nature*, 465(7298):562–564, 2010. doi:10.1038/465562a.

[48] Saskia Decuypere, Jessica Maltha, Stijn Deborggraeve, Nicholas JW Rattray, Guiraud Issa, Kaboré Bérenger, Palpouguini Lompo, Marc C Tahita, Thusitha Ruspasinghe, Malcolm McConville, et al. Towards improving point-of-care diagnosis of non-malaria febrile illness: A metabolomics approach. *PLoS neglected tropical diseases*, 10(3):e0004480, 2016.

[49] William R Beisel. Metabolic response to infection. *Annual review of medicine*, 26(1):9–20, 1975.

[50] Coy D Fitch, Guang-zuan Cai, and James D Shoemaker. A role for linoleic acid in erythrocytes infected with plasmodium berghei. *Biochimica et Biophysica Acta (BBA)-Molecular Basis of Disease*, 1535(1):45–49, 2000.

[51] Michael Maceyka and Sarah Spiegel. Sphingolipid metabolites in inflammatory disease. *Nature*, 510(7503):58, 2014.

[52] Young-Jin Seo, Stephen Alexander, and Bumsuk Hahm. Does cytokine signaling link sphingolipid metabolism to host defense and immunity against virus infections? *Cytokine & growth factor reviews*, 22(1):55–61, 2011.

A Session info

Here is the output of `sessionInfo()` on the system that compiled this vignette:

- R Under development (unstable) (2017-12-31 r73996), `x86_64-pc-linux-gnu`
- Locale: `LC_CTYPE=en_US.UTF-8`, `LC_NUMERIC=C`, `LC_TIME=en_US.UTF-8`, `LC_COLLATE=en_US.UTF-8`, `LC_MONETARY=en_US.UTF-8`, `LC_MESSAGES=C`, `LC_PAPER=en_US.UTF-8`, `LC_NAME=C`, `LC_ADDRESS=C`, `LC_TELEPHONE=C`, `LC_MEASUREMENT=en_US.UTF-8`, `LC_IDENTIFICATION=C`
- Running under: `Debian GNU/Linux 9 (stretch)`
- Matrix products: default
- BLAS: `/usr/lib/openblas-base/libblas.so.3`
- LAPACK: `/usr/lib/libopenblasp-r0.2.19.so`
- Base packages: base, datasets, graphics, grDevices, methods, parallel, stats, stats4, utils
- Other packages: AnnotationDbi 1.41.4, Biobase 2.39.1, BiocGenerics 0.25.3, BiocStyle 2.7.8, diffuStats 0.103.2, expm 0.999-2, FELLA 0.99.3.1, igraph 1.1.2, IRanges 2.13.26, MASS 7.3-48, Matrix 1.2-12, org.Hs.eg.db 3.5.0, plyr 1.8.4, precrec 0.9.1, Rcpp 0.12.14, RcppArmadillo 0.8.300.1.0, RcppParallel 4.3.20, S4Vectors 0.17.33
- Loaded via a namespace (and not attached): assertthat 0.2.0, backports 1.1.2, BiocInstaller 1.29.4, biomaRt 2.35.10, Biostrings 2.47.9, bit 1.1-12, bit64 0.9-7, bitops 1.0-6, blob 1.1.0, bookdown 0.6, colorspace 1.3-2, compiler 3.5.0, curl 3.1, data.table 1.10.4-3, DBI 0.7, devtools 1.13.4, digest 0.6.15, evaluate 0.10.1, ggplot2 2.2.1, GO.db 3.5.0, GOSemSim 2.5.1, grid 3.5.0, gtable 0.2.0, highr 0.6, htmltools 0.3.6, httr 1.3.1, KEGGREST 1.19.2, knitr 1.20, lattice 0.20-35, lazyeval 0.2.1, magrittr 1.5, memoise 1.1.0, munsell 0.4.3, pillar 1.0.1, pkgconfig 2.0.1, png 0.1-7, prettyunits 1.0.2, progress 1.1.2, R6 2.2.2, RCurl 1.95-4.9, rlang 0.2.0, rmarkdown 1.8, rprojroot 1.3-1, RSQLite 2.0, scales 0.5.0, stringi 1.1.6, stringr 1.3.0, tibble 1.4.1, tools 3.5.0, withr 2.1.1, xfun 0.1, XML 3.98-1.9, XVector 0.19.8, yaml 2.1.16, zlibbioc 1.25.0
