# Supplementary material for: FELLA: an R package to enrich metabolomics data
Source: BMC Bioinformatics. 2018 Dec 22;19:538. doi: 10.1186/s12859-018-2487-5 (PMC6303911; doi:10.1186/s12859-018-2487-5)
Supplement: Supplementary file 3 — Case study with FELLA: two datasets on the effect of oxybenzone exposition on gilt-head bream. (PDF 221 kb) [file 12859_2018_2487_MOESM3_ESM.pdf]

An oxybenzone exposition study on gilt-head bream

Sergio Picart-Armada* [*1] ***and Alexandre Perera-Lluna*** [†1]

[1]B2SLab at Polytechnic University of Catalonia

[*]sergi.picart@upc.edu [†]alexandre.perera@upc.edu

September 18, 2018

Package

FELLA 1.1.5

Contents

1 Introduction . . . 2
- 1.1 Building the database . . . 2
- 1.2 Note on reproducibility . . . 3

2 Enrichment analysis on liver tissue . . . 4
- 2.1 Defining the input and running the enrichment . . . 4
- 2.2 Examining the pathways . . . 6
- 2.3 Examining the metabolites . . . 6

3 Enrichment analysis on plasma . . . 7
- 3.1 Defining the input and running the enrichment . . . 7
- 3.2 Examining the pathways . . . 10
- 3.3 Examining the metabolites . . . 11

4 Conclusions . . . 11

5 Reproducibility . . . 11

References . . . 13

1 Introduction

This vignette contains a case study of the effects of environmental contamination on gilt-head bream (*Sparus aurata*) (Ziarrusta et al. 2018). Fish were exposed over 14 days to *oxybenzone* and changes were sought in their brain, liver and plasma using untargeted metabolomics. Samples were processed using Ultra-performance liquid chromatography mass-spectrometry (UHPLC-qOrbitrap MS) in positive and negative modes with both C18 and HILIC separation.

The mortality of exposed fish was not altered, as well as the brain-related metabolites. However, liver and plasma showed perturbations, proving that adverse effects beyond the well-studied hormonal activity were present.

The enrichment procedure implemented in `FELLA` (Picart-Armada et al. 2017) was used in the study for a deeper understanding of the dysregulated metabolites in both tissues.

1.1 Building the database

At the time of publication, the KEGG database (Kanehisa et al. 2016) –upon which `FELLA` is based– did not have pathway annotations for the *Sparus aurata* organism. It is common, however, to use the zebrafish (*Danio rerio*) pathways as a good approximation. KEGG provides pathway annotations for it under the organismal code `dre`, which will be used to build the `FELLA.DATA` object.

```
library(FELLA)

library(igraph)
library(magrittr)

set.seed(1)
# Filter the dre01100 overview pathway, as in the article
graph <- buildGraphFromKEGGREST(
    organism = "dre",
    filter.path = c("01100"))

tmpdir <- paste0(tempdir(), "/my_database")
# Make sure the database does not exist from a former vignette build
# Otherwise the vignette will rise an error
# because FELLA will not overwrite an existing database
unlink(tmpdir, recursive = TRUE)
buildDataFromGraph(
    keggdata.graph = graph,
    databaseDir = tmpdir,
    internalDir = FALSE,
    matrices = "none",
    normality = "diffusion",
    niter = 100)
```

We load the `FELLA.DATA` object to run both analyses:

```
fella.data <- loadKEGGdata(
    databaseDir = tmpdir,
```

```
    internalDir = FALSE,
    loadMatrix = "none"
)
```

Given the 11-month temporal gap between the study and this vignette, small changes to the amount of nodes in each category are expected (see section *2.4 Data handling and statistical analyses* from the study). Please see the Note on reproducibility to understand why.

```
fella.data
## General data:
## - KEGG graph:
##   * Nodes:  9637
##   * Edges:  28864
##   * Density:  0.0003108264
##   * Categories:
##     + pathway [162]
##     + module [179]
##     + enzyme [995]
##     + reaction [4843]
##     + compound [3458]
##   * Size:  5.4 Mb
## - KEGG names are ready.
## -----------------------------
## Hypergeometric test:
## - Matrix not loaded.
## -----------------------------
## Heat diffusion:
## - Matrix not loaded.
## - RowSums are ready.
## -----------------------------
## PageRank:
## - Matrix not loaded.
## - RowSums not loaded.
```

1.2 Note on reproducibility

We want to emphasise that each time this vignette is built, `FELLA` constructs its `FELLA.DATA` object using the most recent version of the KEGG database. KEGG is frequently updated and therefore small changes can take place in the knowledge graph between different releases. The discussion on our findings was written at the date specified in the vignette header and using the KEGG release in the Reproducibility section.

2 Enrichment analysis on liver tissue

2.1 Defining the input and running the enrichment

Table 1 from the main body in (Ziarrusta et al. 2018) contains 5 KEGG identifiers associated to metabolic changes in liver tissue and 12 in plasma. Our first enrichment analysis with `FELLA` will be based on the liver-derived metabolites. Also note that we use the faster `approx = "normality"` approach, whereas the original article uses `approx = "simulation"` with `niter = 15000` This is not only intended to keep the bulding time of this vignette as low as possible, but also to demonstrate that the findings using both statistical approaches are consistent.

```
cpd.liver <- c(
    "C12623",
    "C01179",
    "C05350",
    "C05598",
    "C01586"
)

analysis.liver <- enrich(
    compounds = cpd.liver,
    data = fella.data,
    method = "diffusion",
    approx = "normality")
## No background compounds specified. Default background will be used.
## Running diffusion...
## Computing p-scores through the specified distribution.
## Done.
```

All the metabolites are successfully mapped:

```
analysis.liver %>%
    getInput %>%
    getName(data = fella.data)
## $C12623
## [1] "trans-2,3-Dihydroxycinnamate"
## [2] "(2E)-3-(2,3-Dihydroxyphenyl)prop-2-enoate"
##
## $C01179
## [1] "3-(4-Hydroxyphenyl)pyruvate" "4-Hydroxyphenylpyruvate"
## [3] "p-Hydroxyphenylpyruvic acid"
##
## $C05350
## [1] "2-Hydroxy-3-(4-hydroxyphenyl)propenoate"
## [2] "4-Hydroxy-enol-phenylpyruvate"
##
## $C05598
## [1] "Phenylacetylglycine"
##
## $C01586
## [1] "Hippurate"                "Hippuric acid"
```

```
## [3] "N-Benzoylglycine"        "Benzoylaminoacetic acid"
```

Below is a plot of the reported sub-network using the default parameters. The five metabolites are present and lie within the same connected component.

```
plot(
    analysis.liver,
    method = "diffusion",
    data = fella.data,
    nlimit = 250,
    plotLegend = FALSE)
```

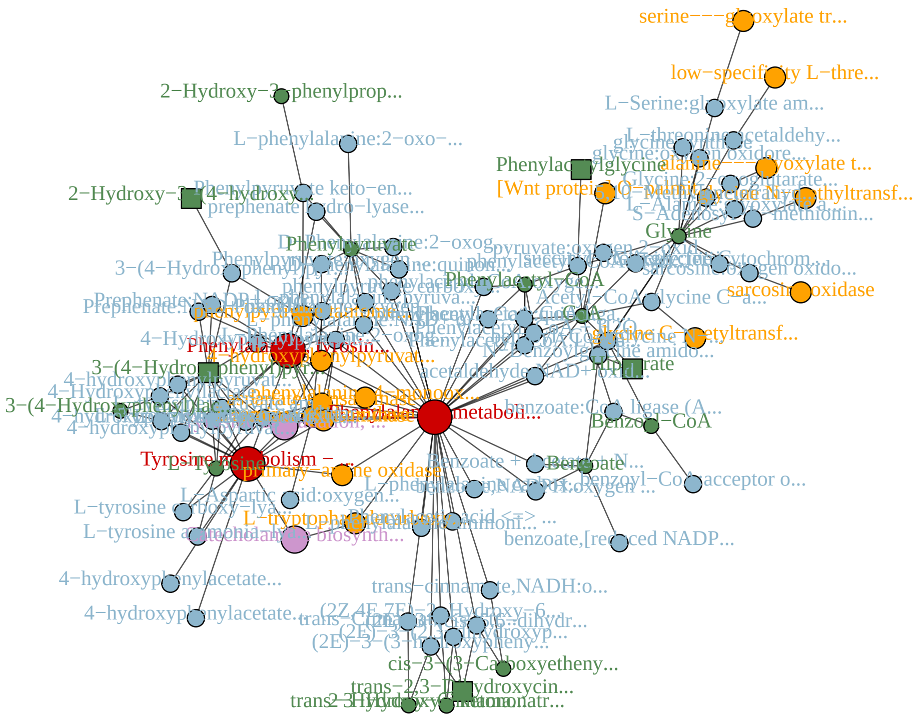

We will examine the igraph object with the reported sub-network and some of its reported entities in tabular format:

```
g.liver <-  generateResultsGraph(
    object = analysis.liver,
    data = fella.data,
    method = "diffusion")

tab.liver <- generateResultsTable(
    object = analysis.liver,
    data = fella.data,
    method = "diffusion")
## Writing diffusion results...
## Done.
```

The reported sub-network contains around 100 nodes and can be manually inquired:

```
g.liver
## IGRAPH d7300ce UNW- 101 166 --
## + attr: organism (g/c), name (v/c), com (v/n), NAME (v/x), entrez
## | (v/x), label (v/c), input (v/l), weight (e/n)
## + edges from d7300ce (vertex names):
##  [1] dre00350--M00042     dre00350--M00044     dre00360--1.13.11.27
##  [4] M00044  --1.13.11.27 dre00360--1.14.16.1  dre00400--1.14.16.1
##  [7] dre00360--1.4.3.2    dre00400--1.4.3.2    M00044  --1.4.3.2
## [10] dre00350--1.4.3.21   dre00360--1.4.3.21   dre00350--2.6.1.1
## [13] dre00360--2.6.1.1    dre00400--2.6.1.1    dre00350--2.6.1.5
## [16] dre00360--2.6.1.5    dre00400--2.6.1.5    dre00360--4.1.1.105
## [19] M00042  --4.1.1.105  dre00350--5.3.2.1    dre00360--5.3.2.1
## + ... omitted several edges
```

2.2 Examining the pathways

Figure 2 from the original study frames the five metabolites in the input around *Phenylalanine metabolism*. We can verify that `FELLA` finds such pathway and two closely related suggestions: *Tyrosine metabolism* and *Phenylalanine, tyrosine and tryptophan biosynthesis*.

```
path.fig2 <- "dre00360" # Phenylalanine metabolism
path.fig2 %in% V(g.liver)$name
## [1] TRUE
```

These are the reported pathways:

```
tab.liver[tab.liver$Entry.type == "pathway", ]
##    KEGG.id Entry.type                                        KEGG.name
## 1 dre00350    pathway    Tyrosine metabolism - Danio rerio (zebrafish)
## 2 dre00360    pathway Phenylalanine metabolism - Danio rerio (zebra...
## 3 dre00400    pathway Phenylalanine, tyrosine and tryptophan biosyn...
##        p.score
## 1 3.519479e-06
## 2 1.000000e-06
## 3 1.030067e-02
```

2.3 Examining the metabolites

Figure 2 also gathers two types of metabolites: metabolites in the input (inside shaded frames) and other contextual metabolites (no frames) that link the input metabolites.

First of all, we can check that all the input metabolites appear in the suggested sub-network. While it's expected that most of the input metabolites appear as relevant, it is an important property of our method, in order to elaborate a sensible biological justification of the experimental differences.

```
cpd.liver %in% V(g.liver)$name
## [1] TRUE TRUE TRUE TRUE TRUE
```

On the other hand, one of the two contextual metabolites is also suggested by FELLA, proving its usefulness to fill the gaps between the input metabolites.

```
cpd.fig2 <- c(
    "C00079", # Phenylalanine
    "C00082"  # Tyrosine
)
cpd.fig2 %in% V(g.liver)$name
## [1] FALSE  TRUE
```

3 Enrichment analysis on plasma

3.1 Defining the input and running the enrichment

As shown in section Defining the input and running the enrichment, 12 KEGG identifiers (one ID is repeated) are related to the experimental changes observed in plasma, which are the starting point of the enrichment:

```
cpd.plasma <- c(
    "C16323",
    "C00740",
    "C08323",
    "C00623",
    "C00093",
    "C06429",
    "C16533",
    "C00740",
    "C06426",
    "C06427",
    "C07289",
    "C01879"
) %>% unique

analysis.plasma <- enrich(
    compounds = cpd.plasma,
    data = fella.data,
    method = "diffusion",
    approx = "normality")
## No background compounds specified. Default background will be used.
## Running diffusion...
## Computing p-scores through the specified distribution.
## Done.
```

The totality of the 11 unique metabolites map to the FELLA.DATA object:

```
analysis.plasma %>%
    getInput %>%
    getName(data = fella.data)
## $C16323
```

```
## [1] "3,6-Nonadienal"
##
## $C00740
## [1] "D-Serine"
##
## $C08323
## [1] "(15Z)-Tetracosenoic acid"  "Nervonic acid"
## [3] "(Z)-15-Tetracosenoic acid"
##
## $C00623
## [1] "sn-Glycerol 1-phosphate" "sn-Gro-1-P"
## [3] "L-Glycerol 1-phosphate"
##
## $C00093
## [1] "sn-Glycerol 3-phosphate" "Glycerophosphoric acid"
## [3] "D-Glycerol 1-phosphate"
##
## $C06429
## [1] "(4Z,7Z,10Z,13Z,16Z,19Z)-Docosahexaenoic acid"
## [2] "4,7,10,13,16,19-Docosahexaenoic acid"
## [3] "Docosahexaenoic acid"
## [4] "Docosahexaenoate"
##
## $C16533
## [1] "13,16-Docosadienoic acid"
## [2] "(13Z,16Z)-Docosa-13,16-dienoic acid"
##
## $C06426
## [1] "(6Z,9Z,12Z)-Octadecatrienoic acid" "6,9,12-Octadecatrienoic acid"
## [3] "gamma-Linolenic acid"              "Gamolenic acid"
##
## $C06427
## [1] "(9Z,12Z,15Z)-Octadecatrienoic acid" "alpha-Linolenic acid"
## [3] "9,12,15-Octadecatrienoic acid"      "Linolenate"
## [5] "alpha-Linolenate"
##
## $C07289
## [1] "Crepenynate"                   "(9Z)-Octadec-9-en-12-ynoate"
## [3] "(Z)-9-Octadecen-12-ynoic acid" "Crepenynic acid"
##
## $C01879
## [1] "5-Oxoproline"                     "Pidolic acid"
## [3] "Pyroglutamic acid"                "5-Pyrrolidone-2-carboxylic acid"
## [5] "Pyroglutamate"                    "5-Oxo-L-proline"
## [7] "L-Pyroglutamic acid"              "L-5-Pyrrolidone-2-carboxylic acid"
```

Again, the reported sub-network consists of a large connected component encompassing most input metabolites:

```
plot(
    analysis.plasma,
```

```
        method = "diffusion",
        data = fella.data,
        nlimit = 250,
        plotLegend = FALSE)
```

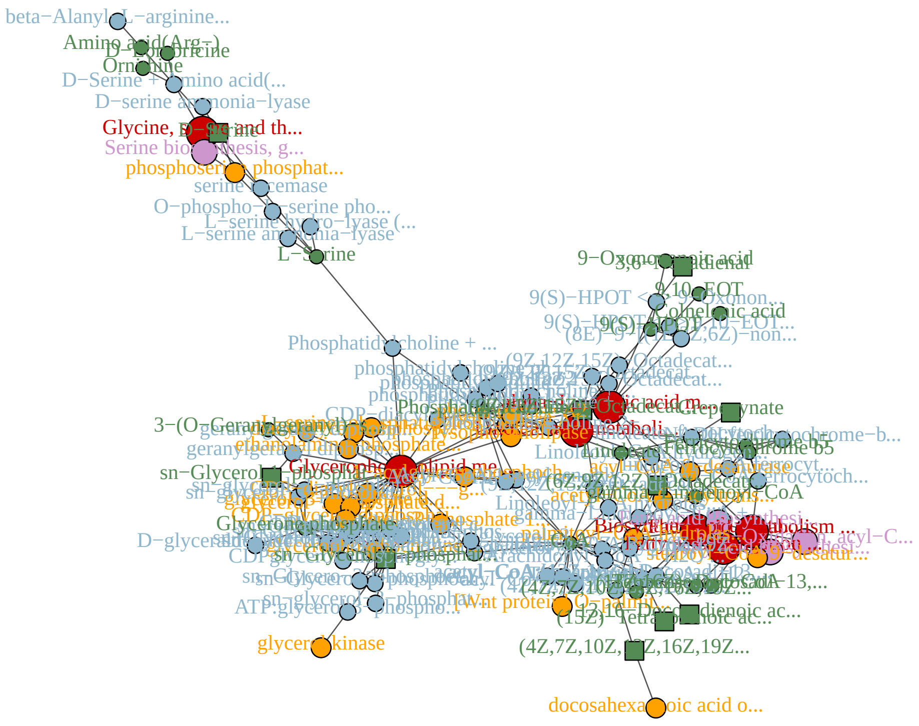

We will export the results as a network and as a table:

```
g.plasma <-  generateResultsGraph(
    object = analysis.plasma,
    data = fella.data,
    method = "diffusion")

tab.plasma <- generateResultsTable(
    object = analysis.plasma,
    data = fella.data,
    method = "diffusion")
## Writing diffusion results...
## Done.
```

The reported sub-network is a bit larger than the one from liver, containing roughly 120 nodes:

```
g.plasma
## IGRAPH f934f4a UNW- 121 197 --
## + attr: organism (g/c), name (v/c), com (v/n), NAME (v/x), entrez
## | (v/x), label (v/c), input (v/l), weight (e/n)
## + edges from f934f4a (vertex names):
##  [1] dre00260--M00020    dre00062--M00085    dre01212--M00085
##  [4] dre01212--M00086    dre00062--M00415    dre01212--M00415
##  [7] dre00564--1.1.1.8   dre01040--1.14.19.1 dre01212--1.14.19.1
```

```
## [10] dre00592--1.14.19.3 dre01040--1.14.19.3 dre01212--1.14.19.3
## [13] dre00564--1.1.5.3   dre00564--2.3.1.15  dre00592--2.3.1.16
## [16] dre01040--2.3.1.16  M00085  --2.3.1.16  dre00564--2.3.1.23
## [19] dre00564--2.7.8.29  dre00564--2.7.8.5   dre00564--3.1.1.32
## + ... omitted several edges
```

3.2 Examining the pathways

Figure 3 from the original study is a holistic view of the affected metabolites found in plasma, based on literature and on an analysis with `FELLA`. The 11 metabolites are depicted within their core metabolic pathways. We will check whether `FELLA` is able to highlight them, by first showing the reported metabolic pathways:

```
tab.plasma[tab.plasma$Entry.type == "pathway", ]
##    KEGG.id Entry.type                                        KEGG.name
## 1 dre00062    pathway Fatty acid elongation - Danio rerio (zebrafis...
## 2 dre00260    pathway Glycine, serine and threonine metabolism - Da...
## 3 dre00564    pathway Glycerophospholipid metabolism - Danio rerio ...
## 4 dre00591    pathway Linoleic acid metabolism - Danio rerio (zebra...
## 5 dre00592    pathway alpha-Linolenic acid metabolism - Danio rerio...
## 6 dre01040    pathway Biosynthesis of unsaturated fatty acids - Dan...
## 7 dre01212    pathway Fatty acid metabolism - Danio rerio (zebrafis...
##        p.score
## 1 1.000000e-06
## 2 6.411148e-06
## 3 2.322060e-05
## 4 3.580059e-02
## 5 1.000000e-06
## 6 1.000000e-06
## 7 4.703301e-04
```

And then comparing against the ones in *Figure 3*:

```
path.fig3 <- c(
    "dre00591", # Linoleic acid metabolism
    "dre01040", # Biosynthesis of unsaturated fatty acids
    "dre00592", # alpha-Linolenic acid metabolism
    "dre00564", # Glycerophospholipid metabolism
    "dre00480", # Glutathione metabolism
    "dre00260"  # Glycine, serine and threonine metabolism
)
path.fig3 %in% V(g.plasma)$name
## [1]  TRUE  TRUE  TRUE  TRUE FALSE  TRUE
```

All of them but *Glutathione metabolism* are recovered, showing how `FELLA` can help gaining perspective on the input metabolites.

3.3 Examining the metabolites

As in the analogous section for liver, we will quantify how many input metabolites, drawn within a shaded frame in *Figure 3*, are reported in the sub-network:

```
cpd.plasma %in% V(g.plasma)$name
##  [1]  TRUE  TRUE  TRUE  TRUE  TRUE  TRUE  TRUE  TRUE  TRUE  TRUE FALSE
```

From the 11 highlighted metabolites, only one is not reported by `FELLA`: *5-Oxo-L-proline*.

Conversely, two out of the three contextual metabolites from the same figure are reported:

```
cpd.fig3 <- c(
    "C01595", # Linoleic acid
    "C00157", # Phosphatidylcholine
    "C00037"  # Glycine
)
cpd.fig3 %in% V(g.plasma)$name
## [1]  TRUE  TRUE FALSE
```

As *Figure 3* shows, the addition of *linoleic acid* and *phosphatidylcholine*, backed up by `FELLA`, helps connecting almost all the metabolites found in blood.

`FELLA` misses *glycine* and, in fact, stays consistent with the pathway (*Glutathione metabolism*) and the input metabolite (*5-Oxo-L-proline*) that it left out from *Figure 3*. The fact that `FELLA` does not suggest such pathway seems to happen at several molecular levels and therefore none of its metabolites are pinpointed.

Even if the glutathione pathway was not reported, `FELLA` can greatly ease the creation of elaborated contextual figures, such as *Figure 3*, by suggesting the intermediate metabolites and the metabolic pathways that link the input compounds.

4 Conclusions

In this vignette, we apply `FELLA` to an untargeted metabolic study of gilt-head bream exposed to an environmental contaminat (oxybenzome). This study is an example of how `FELLA` can be useful for (1) organisms not limited to *Homo sapiens*, and (2) conditions not limited to a specific disease.

On one hand, `FELLA` helps creating complex contextual interpretations of the data, such as the comprehensive *Figure 3* from the original article (Ziarrusta et al. 2018). This material would be challenging to build through regular over-representation analysis of the input metabolites. On the other hand, metabolites and pathways suggested by `FELLA` were also mentioned in the literature and supported the main findings in the study. In particular, it helped identify key processes such as *phenylalanine metabolism*, *alpha-linoleic acid metabolism* and *serine metabolism*, which ultimately pointed to alterations in *oxidative stress*.

5 Reproducibility

This is the result of running `sessionInfo()`

```
sessionInfo()
## R version 3.5.1 (2018-07-02)
## Platform: x86_64-pc-linux-gnu (64-bit)
## Running under: Ubuntu 16.04.5 LTS
##
## Matrix products: default
## BLAS: /usr/lib/atlas-base/atlas/libblas.so.3.0
## LAPACK: /usr/lib/atlas-base/atlas/liblapack.so.3.0
##
## locale:
##  [1] LC_CTYPE=en_US.UTF-8       LC_NUMERIC=C
##  [3] LC_TIME=en_US.UTF-8        LC_COLLATE=en_US.UTF-8
##  [5] LC_MONETARY=en_US.UTF-8    LC_MESSAGES=en_US.UTF-8
##  [7] LC_PAPER=en_US.UTF-8       LC_NAME=C
##  [9] LC_ADDRESS=C               LC_TELEPHONE=C
## [11] LC_MEASUREMENT=en_US.UTF-8 LC_IDENTIFICATION=C
##
## attached base packages:
## [1] parallel  stats4    stats     graphics  grDevices utils     datasets
## [8] methods   base
##
## other attached packages:
##  [1] magrittr_1.5         igraph_1.2.2         KEGGREST_1.20.1
##  [4] org.Mm.eg.db_3.6.0   AnnotationDbi_1.42.1 IRanges_2.14.11
##  [7] S4Vectors_0.18.3     Biobase_2.40.0       BiocGenerics_0.26.0
## [10] FELLA_1.1.5          BiocStyle_2.8.2
##
## loaded via a namespace (and not attached):
##  [1] Rcpp_0.12.18         highr_0.7            BiocInstaller_1.30.0
##  [4] compiler_3.5.1       plyr_1.8.4           XVector_0.20.0
##  [7] tools_3.5.1          zlibbioc_1.26.0      bit_1.1-14
## [10] digest_0.6.17        RSQLite_2.1.1        evaluate_0.11
## [13] memoise_1.1.0        lattice_0.20-35      pkgconfig_2.0.2
## [16] png_0.1-7            Matrix_1.2-14        DBI_1.0.0
## [19] curl_3.2             yaml_2.2.0           xfun_0.3
## [22] withr_2.1.2          httr_1.3.1           stringr_1.3.1
## [25] knitr_1.20           Biostrings_2.48.0    devtools_1.13.6
## [28] bit64_0.9-7          rprojroot_1.3-2      grid_3.5.1
## [31] R6_2.2.2             rmarkdown_1.10       bookdown_0.7
## [34] blob_1.1.1           backports_1.1.2      htmltools_0.3.6
## [37] tinytex_0.8          stringi_1.2.4
```

KEGG version:

```
cat(getInfo(fella.data))
## T01004           Danio rerio (zebrafish) KEGG Genes Database
## dre              Release 87.0+/09-20, Sep 18
##                  Kanehisa Laboratories
##                  30,640 entries
##
## linked db        pathway
```

```
##                    brite
##                    module
##                    ko
##                    genome
##                    enzyme
##                    ncbi-geneid
##                    ncbi-proteinid
##                    uniprot
```

Date of generation:

```
date()
## [1] "Thu Sep 20 16:27:39 2018"
```

Image of the workspace (for submission):

```
tempfile(pattern = "vignette_dre_", fileext = ".RData") %T>%
    message("Saving workspace to ", .) %>%
    save.image(compress = "xz")
## Saving workspace to /tmp/RtmpQORpuY/vignette_dre_2e36ca31e32.RData
```

References

Kanehisa, Minoru, Miho Furumichi, Mao Tanabe, Yoko Sato, and Kanae Morishima. 2016. "KEGG: New Perspectives on Genomes, Pathways, Diseases and Drugs." *Nucleic Acids Research* 45 (D1). Oxford University Press: D353–D361.

Picart-Armada, Sergio, Francesc Fernández-Albert, Maria Vinaixa, Miguel A Rodríguez, Suvi Aivio, Travis H Stracker, Oscar Yanes, and Alexandre Perera-Lluna. 2017. "Null Diffusion-Based Enrichment for Metabolomics Data." *PloS One* 12 (12). Public Library of Science: e0189012.

Ziarrusta, Haizea, Leire Mijangos, Sergio Picart-Armada, Mireia Irazola, Alexandre Perera-Lluna, Aresatz Usobiaga, Ailette Prieto, Nestor Etxebarria, Maitane Olivares, and Olatz Zuloaga. 2018. "Non-Targeted Metabolomics Reveals Alterations in Liver and Plasma of Gilt-Head Bream Exposed to Oxybenzone." *Chemosphere* 211. Elsevier: 624–31.
