# Supplementary material for: FELLA: an R package to enrich metabolomics data
Source: BMC Bioinformatics. 2018 Dec 22;19:538. doi: 10.1186/s12859-018-2487-5 (PMC6303911; doi:10.1186/s12859-018-2487-5)
Supplement: Supplementary file 4 — Case study with FELLA: a multi-omic mouse model of non-alcoholic fatty liver disease. (PDF 235 kb) [file 12859_2018_2487_MOESM4_ESM.pdf]

A fatty liver study on Mus musculus

Sergio Picart-Armada [*1] ***and Alexandre Perera-Lluna*** [†1]

[1]B2SLab at Polytechnic University of Catalonia

[*]sergi.picart@upc.edu [†]alexandre.perera@upc.edu

September 17, 2018

Package

FELLA 1.1.5

Contents

1 Introduction . . . 2
  1.1 Building the database . . . 2
  1.2 Note on reproducibility . . . 3
2 Enrichment analysis . . . 4
  2.1 Defining the input and running the enrichment . . . 4
  2.2 Examining the metabolites . . . 6
  2.3 Examining the genes . . . 8
3 Conclusions . . . 12
4 Reproducibility . . . 13
References . . . 14

1 Introduction

This vignette shows the utility of the FELLA package, which is based in a statistically normalised diffusion process (Picart-Armada et al. 2017), on non-human organisms. In particular, we will work on a multi-omic *Mus musculus* study. The original study (Gogiashvili et al. 2017) presents a mouse model of the non-alcoholic fatty liver disease (NAFLD). Metabolites in liver tissue from leptin-deficient *ob/ob* mice and wild type mice were compared using Nuclear Magnetic Resonance (NMR). Afterwards, quantitative real-time polymerase chain reaction (qRT-PCR) helped identify changes at the gene expression level. Finally, biological mechanisms behind NAFLD were elucidated by leveraging the data from both omics.

1.1 Building the database

The first step is to build the FELLA.DATA object for the mmu organism from the KEGG database (Kanehisa et al. 2016).

```
library(FELLA)
library(org.Mm.eg.db)
library(KEGGREST)

library(igraph)
library(magrittr)

set.seed(1)
# Filter overview pathways
graph <- buildGraphFromKEGGREST(
    organism = "mmu",
    filter.path = c("01100", "01200", "01210", "01212", "01230"))

tmpdir <- paste0(tempdir(), "/my_database")
# Mke sure the database does not exist from a former vignette build
# Otherwise the vignette will rise an error
# because FELLA will not overwrite an existing database
unlink(tmpdir, recursive = TRUE)
buildDataFromGraph(
    keggdata.graph = graph,
    databaseDir = tmpdir,
    internalDir = FALSE,
    matrices = "none",
    normality = "diffusion",
    niter = 100)
```

We load the FELLA.DATA object and two mappings (from gene symbol to entrez identifiers, and from enzyme EC numbers to their annotated entrez genes).

```
alias2entrez <- as.list(org.Mm.eg.db::org.Mm.egSYMBOL2EG)
entrez2ec <- KEGGREST::keggLink("enzyme", "mmu")
entrez2path <- KEGGREST::keggLink("pathway", "mmu")

fella.data <- loadKEGGdata(
```

```
    databaseDir = tmpdir,
    internalDir = FALSE,
    loadMatrix = "none"
)
```

Summary of the database:

```
fella.data
## General data:
## - KEGG graph:
##   * Nodes:  9909
##   * Edges:  31127
##   * Density:  0.0003170454
##   * Categories:
##     + pathway [316]
##     + module [185]
##     + enzyme [1107]
##     + reaction [4843]
##     + compound [3458]
##   * Size:  5.6 Mb
## - KEGG names are ready.
## -----------------------------
## Hypergeometric test:
## - Matrix not loaded.
## -----------------------------
## Heat diffusion:
## - Matrix not loaded.
## - RowSums are ready.
## -----------------------------
## PageRank:
## - Matrix not loaded.
## - RowSums not loaded.
```

In addition, we will store the ids of all the metabolites, reactions and enzymes in the database:

```
id.cpd <- getCom(fella.data, level = 5, format = "id") %>% names
id.rx <- getCom(fella.data, level = 4, format = "id") %>% names
id.ec <- getCom(fella.data, level = 3, format = "id") %>% names
```

1.2 Note on reproducibility

We want to emphasise that `FELLA` builds its `FELLA.DATA` object using the most recent version of the KEGG database. KEGG is frequently updated and therefore small changes can take place in the knowledge graph between different releases. The discussion on our findings was written at the date specified in the vignette header and using the KEGG release in the Reproducibility section.

2 Enrichment analysis

2.1 Defining the input and running the enrichment

Table 2 from the main body in (Gogiashvili et al. 2017) contains six metabolites that show significant changes between the experimental classes by a univariate test followed by multiple test correction. These are the start of our enrichment analysis:

```
cpd.nafld <- c(
    "C00020", # AMP
    "C00719", # Betaine
    "C00114", # Choline
    "C00037", # Glycine
    "C00160", # Glycolate
    "C01104"  # Trimethylamine-N-oxide
)

analysis.nafld <- enrich(
    compounds = cpd.nafld,
    data = fella.data,
    method = "diffusion",
    approx = "normality")
## No background compounds specified. Default background will be used.
## Warning in defineCompounds(compounds = compounds, compoundsBackground =
## compoundsBackground, : Some compounds were introduced as affected but they
## do not belong to the background. These compounds will be excluded from the
## analysis. Use 'getExcluded' to see them.
## Running diffusion...
## Computing p-scores through the specified distribution.
## Done.
```

Five compounds are successfully mapped to the graph object:

```
analysis.nafld %>%
    getInput %>%
    getName(data = fella.data)
## $C00020
## [1] "AMP"                          "Adenosine 5'-monophosphate"
## [3] "Adenylic acid"                "Adenylate"
## [5] "5'-AMP"                       "5'-Adenylic acid"
## [7] "5'-Adenosine monophosphate" "Adenosine 5'-phosphate"
##
## $C00037
## [1] "Glycine"          "Aminoacetic acid" "Gly"
##
## $C00114
## [1] "Choline"     "Bilineurine"
##
## $C00160
## [1] "Glycolate"          "Glycolic acid"      "Hydroxyacetic acid"
##
```

```
## $C00719
## [1] "Betaine"                    "Trimethylaminoacetate"
## [3] "Glycine betaine"            "N,N,N-Trimethylglycine"
## [5] "Trimethylammonioacetate"
```

Likewise, one compound does not map:

```
getExcluded(analysis.nafld)
## [1] "C01104"
```

The highlighted subgraph with the default parameters has the following appeareance, with large connected components that involve the metabolites in the input:

```
plot(
    analysis.nafld,
    method = "diffusion",
    data = fella.data,
    nlimit = 250,
    plotLegend = FALSE)
```

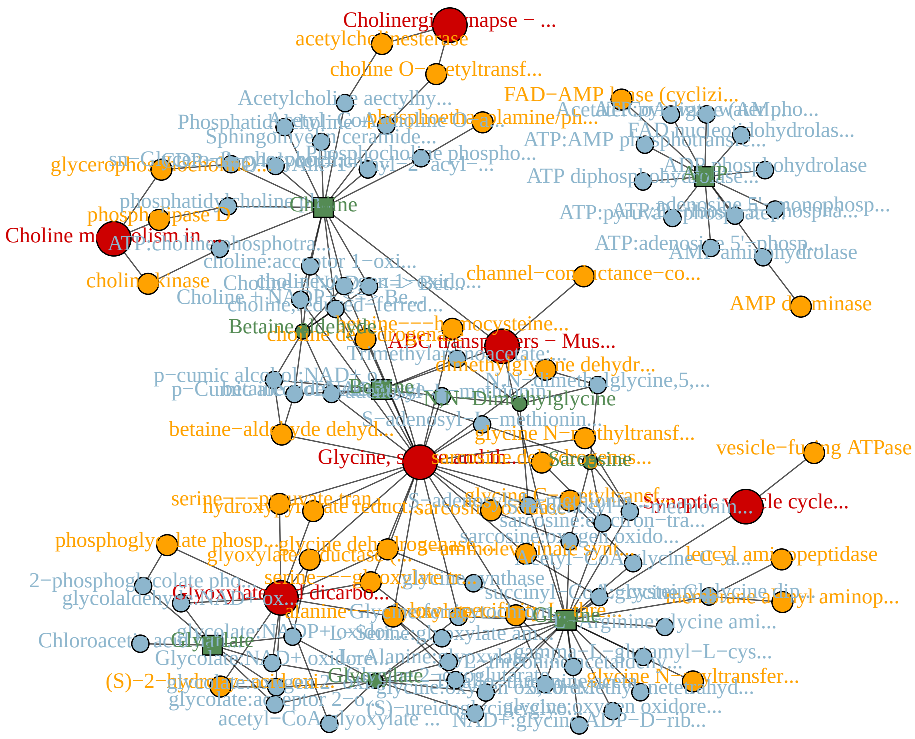

We will also extract all the p-scores and the suggested sub-network for further analysis:

```
g.nafld <-  generateResultsGraph(
    object = analysis.nafld,
    data = fella.data,
    method = "diffusion")

pscores.nafld <- getPscores(
```

```
    object = analysis.nafld,
    method = "diffusion")
```

2.2 Examining the metabolites

2.2.1 From Table 2

The authors find 5 extra metabolites in *Table 2* that are significant at $p < 0.05$ but do not appear after thresholding the false discovery rate at 5%. Such metabolites, highlighted in italics but without an asterisk, are also relevant and play a role in their discussion. We will examine how `FELLA` prioritises such metabolites:

```
cpd.nafld.suggestive <- c(
    "C00008", # ADP
    "C00791", # Creatinine
    "C00025", # Glutamate
    "C01026", # N,N-dimethylglycine
    "C00079", # Phenylalanine
    "C00299"  # Uridine
)
getName(cpd.nafld.suggestive, data = fella.data)
## $C00008
## [1] "ADP"                      "Adenosine 5'-diphosphate"
##
## $C00791
## [1] "Creatinine"             "1-Methylglycocyamidine"
##
## $C00025
## [1] "L-Glutamate"       "L-Glutamic acid"   "L-Glutaminic acid"
## [4] "Glutamate"
##
## $C01026
## [1] "N,N-Dimethylglycine" "Dimethylglycine"
##
## $C00079
## [1] "L-Phenylalanine"
## [2] "(S)-alpha-Amino-beta-phenylpropionic acid"
##
## $C00299
## [1] "Uridine"
```

When checking if any of these metabolites are found in the reported sub-network, we find that `C01026` is already reported:

```
V(g.nafld)$name %>%
    intersect(cpd.nafld.suggestive) %>%
    getName(data = fella.data)
## $C01026
## [1] "N,N-Dimethylglycine" "Dimethylglycine"
```

Abbreviated as **DMG** in their study, N,N-Dimethylglycine is a cornerstone of their findings. It is reported in Figure 6a as part of the folate-independent remethylation to explain the metabolic changes observed in the *ob/ob* mice. **DMG** is also mentioned in the conclusions as part of one of the most prominent alterations found in the study: a reduced conversion of betaine to **DMG**.

2.2.2 From Figure 6a

Figure 6a contains the metabolic context of the observed alterations, with processes such as transsulfuration and folate-dependent remethylation. These were identified with the help of gene expression analysis. We will now check for coincidences between the metabolites in *Figure 6a*, excluding choline and betaine for being in the input and DMG since it was already discussed.

```
cpd.new.fig6 <- c(
    "C00101", # THF
    "C00440", # 5-CH3-THF
    "C00143", # 5,10-CH3-THF
    "C00073", # Methionine
    "C00019", # SAM
    "C00021", # SAH
    "C00155", # Homocysteine
    "C02291", # Cystathione
    "C00097"  # Cysteine
)
getName(cpd.new.fig6, data = fella.data)
## $C00101
## [1] "Tetrahydrofolate"          "5,6,7,8-Tetrahydrofolate"
## [3] "Tetrahydrofolic acid"      "THF"
## [5] "(6S)-Tetrahydrofolate"     "(6S)-Tetrahydrofolic acid"
## [7] "(6S)-THFA"
##
## $C00440
## [1] "5-Methyltetrahydrofolate"
##
## $C00143
## [1] "5,10-Methylenetetrahydrofolate"
## [2] "(6R)-5,10-Methylenetetrahydrofolate"
## [3] "5,10-Methylene-THF"
##
## $C00073
## [1] "L-Methionine"                      "Methionine"
## [3] "L-2-Amino-4methylthiobutyric acid"
##
## $C00019
## [1] "S-Adenosyl-L-methionine" "S-Adenosylmethionine"
## [3] "AdoMet"                  "SAM"
##
## $C00021
## [1] "S-Adenosyl-L-homocysteine" "S-Adenosylhomocysteine"
##
```

```
## $C00155
## [1] "L-Homocysteine"                   "L-2-Amino-4-mercaptobutyric acid"
## [3] "Homocysteine"
##
## $C02291
## [1] "L-Cystathionine"
##
## $C00097
## [1] "L-Cysteine"                        "L-2-Amino-3-mercaptopropionic acid"
```

This time, there are no coincidences with the reported sub-network:

```
cpd.new.fig6 %in% V(g.nafld)$name
## [1] FALSE FALSE FALSE FALSE FALSE FALSE FALSE FALSE FALSE
```

However, we can further inquire whether the p-scores of such metabolites tend to be low among all the metabolites in the whole network from the `fella.data` object.

```
wilcox.test(
    x = pscores.nafld[cpd.new.fig6], # metabolites from fig6
    y = pscores.nafld[setdiff(id.cpd, cpd.new.fig6)], # rest of metabolites
    alternative = "less")
##
##  Wilcoxon rank sum test with continuity correction
##
## data:  pscores.nafld[cpd.new.fig6] and pscores.nafld[setdiff(id.cpd, cpd.new.fig6)]
## W = 651, p-value = 3.333e-07
## alternative hypothesis: true location shift is less than 0
```

The test is indeed significant – despite `FELLA` does not directly report such metabolites, its metabolite ranking supports the claims by the authors.

2.3 Examining the genes

2.3.1 Cbs

The authors complement the metabolomic profilings with a differential gene expression study. One of the main findings is a change of *Cbs* expression levels. To link *Cbs* to the enrichment from `FELLA`, we will first map it to its EC number, *4.2.1.22* at the time of writing:

```
ec.cbs <- entrez2ec[[paste0("mmu:", alias2entrez[["Cbs"]])]] %>%
    gsub(pattern = "ec:", replacement = "")

getName(fella.data, ec.cbs)
## $`4.2.1.22`
## [1] "cystathionine beta-synthase"
## [2] "serine sulfhydrase"
## [3] "beta-thionase"
## [4] "methylcysteine synthase"
## [5] "cysteine synthase (incorrect)"
```

```
## [6] "serine sulfhydrylase"
## [7] "L-serine hydro-lyase (adding homocysteine)"
```

In *Figure 6a*, the reaction linked to *Cbs* and catalysed by the enzyme *4.2.1.22* has the KEGG identifier *R01290*.

```
rx.cbs <- "R01290"

getName(fella.data, rx.cbs)
## $R01290
## [1] "L-serine hydro-lyase (adding homocysteine"
## [2] "L-cystathionine-forming)"
## [3] "L-Serine + L-Homocysteine <=> L-Cystathionine + H2O"
```

As shown in *Figure 6a*, *Cbs* is not directly linked to the metabolites found through NMR, and nor the reaction neither the enzyme are suggested by `FELLA`:

```
c(rx.cbs, ec.cbs) %in% V(g.nafld)$name
## [1] FALSE FALSE
```

However, both of them have a relatively low p-score in their respective categories. This can be seen through the proportion of enzymes (resp. reactions) that show a p-score as low or lower than *4.2.1.22* (resp. *R01290*)

```
# enzyme
pscores.nafld[ec.cbs]
##  4.2.1.22
## 0.3654596
mean(pscores.nafld[id.ec] <= pscores.nafld[ec.cbs])
## [1] 0.163505

# reaction
pscores.nafld[rx.cbs]
##    R01290
## 0.2382633
mean(pscores.nafld[id.rx] <= pscores.nafld[rx.cbs])
## [1] 0.02808177
```

It's not surprising that none of them is directly reported, because none of the metabolites participating in the reaction is found in the input. The main evidence for finding *Cbs* is gene expression, and our approach gives indirect hints of this connection.

2.3.2 Bhmt

The alteration of *Bhmt* activity is related to the downregulation of *Cbs*. Despite not finding evidence of change in *Bhmt* expression, the authors argue that its inhibition would explain the increased betaine-to-DMG ratio in *ob/ob* mice. Such claim is also backed up by prior studies. To find out the role of *Cbs* in our analysis, we will again map it to its EC number, *2.1.1.5*:

```
ec.bhmt <- entrez2ec[[paste0("mmu:", alias2entrez[["Bhmt"]])]] %>%
    gsub(pattern = "ec:", replacement = "")
```

```
getName(fella.data, ec.bhmt)
## $`2.1.1.5`
## [1] "betaine---homocysteine S-methyltransferase"
## [2] "betaine-homocysteine methyltransferase"
## [3] "betaine-homocysteine transmethylase"
```

This time, `FELLA` not only reports it, but also its associated reaction *R02821* (represented by an arrow in *Figure 6a*) and both of its metabolites. While **betaine** was already an input metabolite, **DMG** was a novel finding as discussed earlier

```
ec.bhmt %in% V(g.nafld)$name
## [1] TRUE
"R02821" %in% V(g.nafld)$name
## [1] TRUE
```

This illustrates how `FELLA` can translate knowledge from dysregulated metabolites to other molecular levels, such as reactions and enzymes.

2.3.3 Slc22a5

The decrease of *Bhmt* activity is later connected to the upregulation of *Slc22a5*, also proved within the original study. However, *Slc22a5* does not map to any EC number and therefore it cannot be found through `FELLA`:

```
entrez.slc22a5 <- alias2entrez[["Slc22a5"]]
entrez.slc22a5 %in% names(entrez2ec)
## [1] FALSE
```

As a matter of fact, the only connection that can be found from KEGG is the role of *Slc22a5* in the *Choline metabolism in cancer* pathway.

```
path.slc22a5 <- entrez2path[paste0("mmu:", entrez.slc22a5)] %>%
    gsub(pattern = "path:", replacement = "")

getName(fella.data, path.slc22a5)
## $mmu05231
## [1] "Choline metabolism in cancer - Mus musculus (mouse)"
```

Coincidentally, this pathway is reported in the sub-graph:

```
path.slc22a5 %in% V(g.nafld)$name
## [1] TRUE
```

2.3.4 Genes from Figure 3

We also examined if genes from *Table 3* were reachable in our analysis. These five literature-derived genes were experimentally confirmed to show gene expression changes, in order to prove that RNA extracted after the metabolomic profiling was still reliable for further transcriptomic analyses. However, only *Scd2* maps to an enzymatic family:

```
symbol.fig3 <- c(
    "Cd36",
    "Scd2",
    "Apoa4",
    "Lcn2",
    "Apom")

entrez.fig3 <- alias2entrez[symbol.fig3] %>% unlist %>% unique
ec.fig3 <- entrez2ec[paste0("mmu:", entrez.fig3)] %T>%
    print %>%
    unlist %>%
    unique %>%
    na.omit %>%
    gsub(pattern = "ec:", replacement = "")
##          <NA>      mmu:20250            <NA>            <NA>            <NA>
##            NA "ec:1.14.19.1"              NA              NA              NA

getName(fella.data, ec.fig3)
## $`1.14.19.1`
## [1] "stearoyl-CoA 9-desaturase"
## [2] "Delta9-desaturase"
## [3] "acyl-CoA desaturase"
## [4] "fatty acid desaturase"
## [5] "stearoyl-CoA, hydrogen-donor:oxygen oxidoreductase"
```

Such family is not reported in our sub-graph

```
ec.fig3 %in% V(g.nafld)$name
## [1] FALSE
```

In addition, its p-score is high compared to other enzymes

```
pscores.nafld[ec.fig3]
## 1.14.19.1
## 0.6208381
mean(pscores.nafld[id.ec] <= pscores.nafld[ec.fig3])
## [1] 0.902439
```

The fact that only one gene mapped to an EC number hinders the potential findings using `FELLA`, and is probably the main reason why `FELLA` missed *Scd2*. In addition, `FELLA` defines a knowledge model that offers simplicity and interpretability, at the cost of introducing limitations on how sophisticated its findings can be.

2.3.5 Genes from Table S2

In parallel with the original study, and cited within its main body, gene array expression data was collected (Godoy et al. 2016) and its hits are included in the supplementary *Table S2* from (Gogiashvili et al. 2017). These genes include the already discussed *Cbs*. We will attempt to link the genes marked as significantly changed to our reported sub-network. In contrast with *Figure 3*, all the genes map to an EC number:

```
symbol.tableS2 <- c(
    "Mat1a",
    "Ahcyl2",
    "Cbs",
    "Mat2b",
    "Mtr")
entrez.tableS2 <- alias2entrez[symbol.tableS2] %>% unlist %>% unique
ec.tableS2 <- entrez2ec[paste0("mmu:", entrez.tableS2)] %T>%
    print %>%
    unlist %>%
    unique %>%
    na.omit %>%
    gsub(pattern = "ec:", replacement = "")
##     mmu:11720     mmu:74340     mmu:12411    mmu:108645    mmu:238505
##  "ec:2.5.1.6"  "ec:3.3.1.1" "ec:4.2.1.22"  "ec:2.5.1.6" "ec:2.1.1.13"
```

None of these EC families are reported in the sub-graph:

```
ec.tableS2 %in% V(g.nafld)$name
## [1] FALSE FALSE FALSE FALSE
```

But in this case, their scores tend to be lower than the rest of enzymes:

```
wilcox.test(
    x = pscores.nafld[ec.tableS2], # enzymes from table S2
    y = pscores.nafld[setdiff(id.ec, ec.tableS2)], # rest of enzymes
    alternative = "less")
##
##  Wilcoxon rank sum test with continuity correction
##
## data:  pscores.nafld[ec.tableS2] and pscores.nafld[setdiff(id.ec, ec.tableS2)]
## W = 1013, p-value = 0.03086
## alternative hypothesis: true location shift is less than 0
```

These findings suggest that if the annotation database is complete enough, `FELLA` can provide a meaningful priorisisation of the enzymes surrounding the affected metabolites.

3 Conclusions

`FELLA` has been used to give a biological meaning to a list of 6 metabolites extracted from a multi-omic study of a mouse model of NAFLD. It has been able to reproduce some findings at the metabolite and gene expression levels, whereas most of the times missed entities would still present a low ranking compared to their background in the database.
The bottom line from our analysis in the present vignette is that `FELLA` not only works on human studies, but also generalises to animal models.

4 Reproducibility

This is the result of running `sessionInfo()`

```
sessionInfo()
## R version 3.5.1 (2018-07-02)
## Platform: x86_64-pc-linux-gnu (64-bit)
## Running under: Ubuntu 16.04.5 LTS
##
## Matrix products: default
## BLAS: /usr/lib/atlas-base/atlas/libblas.so.3.0
## LAPACK: /usr/lib/atlas-base/atlas/liblapack.so.3.0
##
## locale:
##  [1] LC_CTYPE=en_US.UTF-8       LC_NUMERIC=C
##  [3] LC_TIME=en_US.UTF-8        LC_COLLATE=en_US.UTF-8
##  [5] LC_MONETARY=en_US.UTF-8    LC_MESSAGES=en_US.UTF-8
##  [7] LC_PAPER=en_US.UTF-8       LC_NAME=C
##  [9] LC_ADDRESS=C               LC_TELEPHONE=C
## [11] LC_MEASUREMENT=en_US.UTF-8 LC_IDENTIFICATION=C
##
## attached base packages:
## [1] parallel  stats4    stats     graphics  grDevices utils     datasets
## [8] methods   base
##
## other attached packages:
##  [1] magrittr_1.5         igraph_1.2.2         KEGGREST_1.20.1
##  [4] org.Mm.eg.db_3.6.0   AnnotationDbi_1.42.1 IRanges_2.14.11
##  [7] S4Vectors_0.18.3     Biobase_2.40.0       BiocGenerics_0.26.0
## [10] FELLA_1.1.5          BiocStyle_2.8.2
##
## loaded via a namespace (and not attached):
##  [1] Rcpp_0.12.18         BiocInstaller_1.30.0 compiler_3.5.1
##  [4] plyr_1.8.4           XVector_0.20.0       tools_3.5.1
##  [7] zlibbioc_1.26.0      bit_1.1-14           digest_0.6.17
## [10] RSQLite_2.1.1        evaluate_0.11        memoise_1.1.0
## [13] lattice_0.20-35      pkgconfig_2.0.2      png_0.1-7
## [16] Matrix_1.2-14        DBI_1.0.0            curl_3.2
## [19] yaml_2.2.0           xfun_0.3             withr_2.1.2
## [22] httr_1.3.1           stringr_1.3.1        knitr_1.20
## [25] Biostrings_2.48.0    devtools_1.13.6      bit64_0.9-7
## [28] rprojroot_1.3-2      grid_3.5.1           R6_2.2.2
## [31] rmarkdown_1.10       bookdown_0.7         blob_1.1.1
## [34] backports_1.1.2      htmltools_0.3.6      stringi_1.2.4
```

KEGG version:

```
cat(getInfo(fella.data))
## T01002           Mus musculus (mouse) KEGG Genes Database
## mmu              Release 87.0+/09-20, Sep 18
##                  Kanehisa Laboratories
```

```
##                  36,154 entries
##
## linked db        pathway
##                  brite
##                  module
##                  ko
##                  genome
##                  mgi
##                  enzyme
##                  ncbi-geneid
##                  ncbi-proteinid
##                  uniprot
```

Date of generation:

```
date()
## [1] "Thu Sep 20 16:25:38 2018"
```

Image of the workspace (for submission):

```
tempfile(pattern = "vignette_mmu_", fileext = ".RData") %T>%
    message("Saving workspace to ", .) %>%
    save.image(compress = "xz")
## Saving workspace to /tmp/RtmpQ0RpuY/vignette_mmu_2e33f36219d.RData
```

References

Godoy, Patricio, Agata Widera, Wolfgang Schmidt-Heck, Gisela Campos, Christoph Meyer, Cristina Cadenas, Raymond Reif, et al. 2016. "Gene Network Activity in Cultivated Primary Hepatocytes Is Highly Similar to Diseased Mammalian Liver Tissue." *Archives of Toxicology* 90 (10). Springer: 2513–29.

Gogiashvili, Mikheil, Karolina Edlund, Kathrin Gianmoena, Rosemarie Marchan, Alexander Brik, Jan T Andersson, Jörg Lambert, et al. 2017. "Metabolic Profiling of Ob/ob Mouse Fatty Liver Using HR-MAS 1 H-NMR Combined with Gene Expression Analysis Reveals Alterations in Betaine Metabolism and the Transsulfuration Pathway." *Analytical and Bioanalytical Chemistry* 409 (6). Springer: 1591–1606.

Kanehisa, Minoru, Miho Furumichi, Mao Tanabe, Yoko Sato, and Kanae Morishima. 2016. "KEGG: New Perspectives on Genomes, Pathways, Diseases and Drugs." *Nucleic Acids Research* 45 (D1). Oxford University Press: D353–D361.

Picart-Armada, Sergio, Francesc Fernández-Albert, Maria Vinaixa, Miguel A Rodríguez, Suvi Aivio, Travis H Stracker, Oscar Yanes, and Alexandre Perera-Lluna. 2017. "Null Diffusion-Based Enrichment for Metabolomics Data." *PloS One* 12 (12). Public Library of Science: e0189012.
