# Supplementary figures and images for: FELLA: an R package to enrich metabolomics data
Source: BMC Bioinformatics. 2018 Dec 22;19:538. doi: 10.1186/s12859-018-2487-5 (PMC6303911; doi:10.1186/s12859-018-2487-5)

## Nodes reported in the 'malaria' dataset

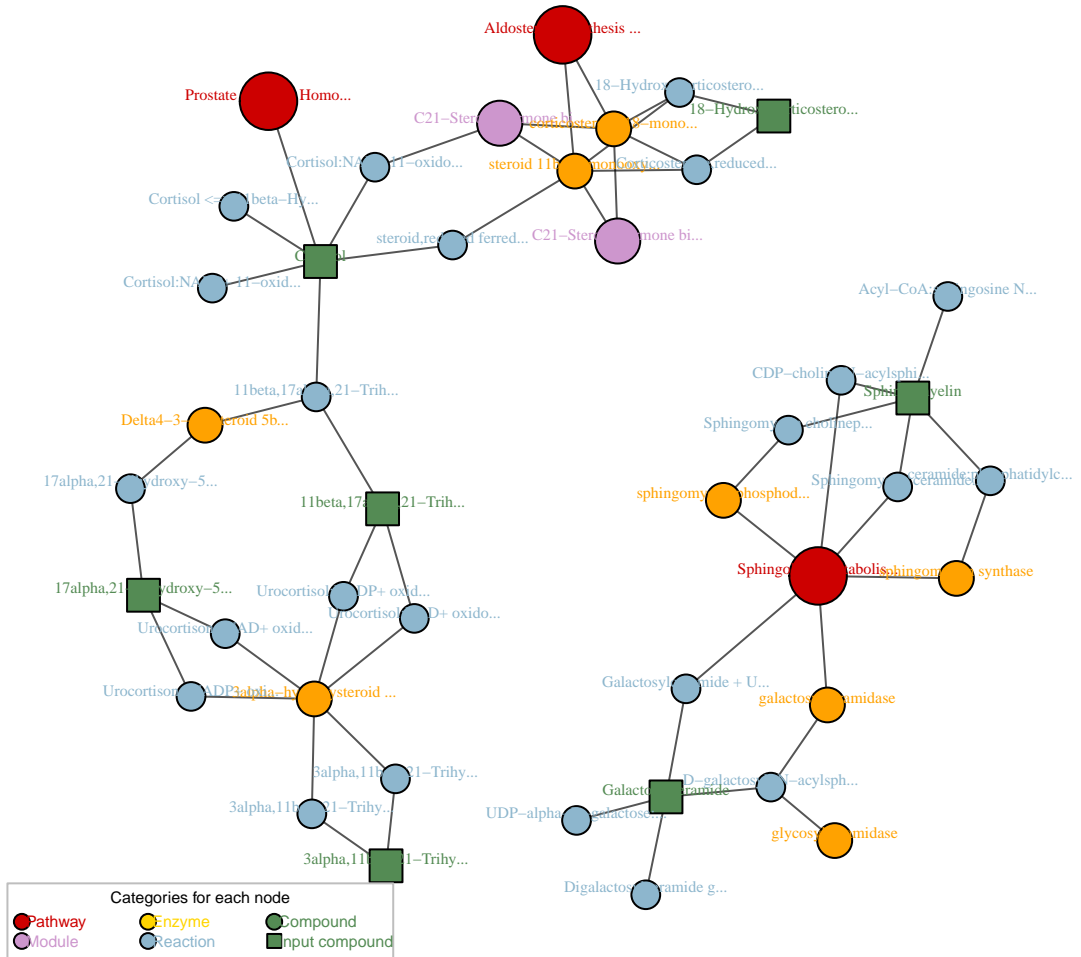

Node limit: 50

Supplement: Supplementary file 5 — Descriptive files on the three human datasets: a summary of the inputs (descriptive_input.csv), input and reported subgraph in each dataset (dataset_input.csv, dataset_subgraph.csv and dataset_subgraph.pdf), hits discussed in the results section (descriptive_hits.csv). Also contains the database object (fella_data.RData) and metadata about the database (info_fella_data.txt), the KEGG version (info_kegg.txt) and the R session (info_session.txt). (ZIP 525 kb) [file 12859_2018_2487_MOESM5_ESM.zip › fella_sm/malaria_subgraph.pdf]

## Nodes reported in the 'ovarian' dataset

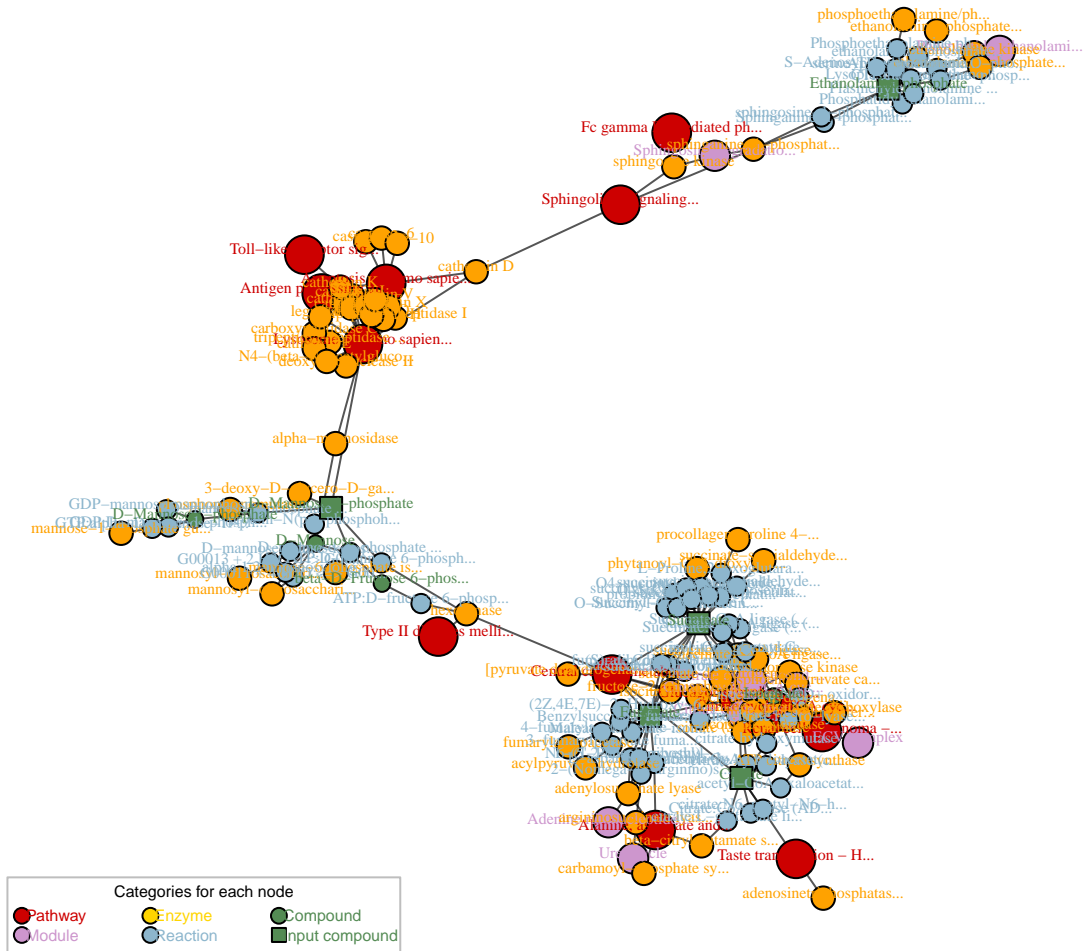

Node limit: 150

Supplement: Supplementary file 5 — Descriptive files on the three human datasets: a summary of the inputs (descriptive_input.csv), input and reported subgraph in each dataset (dataset_input.csv, dataset_subgraph.csv and dataset_subgraph.pdf), hits discussed in the results section (descriptive_hits.csv). Also contains the database object (fella_data.RData) and metadata about the database (info_fella_data.txt), the KEGG version (info_kegg.txt) and the R session (info_session.txt). (ZIP 525 kb) [file 12859_2018_2487_MOESM5_ESM.zip › fella_sm/ovarian_subgraph.pdf]

## Nodes reported in the 'epithelial' dataset

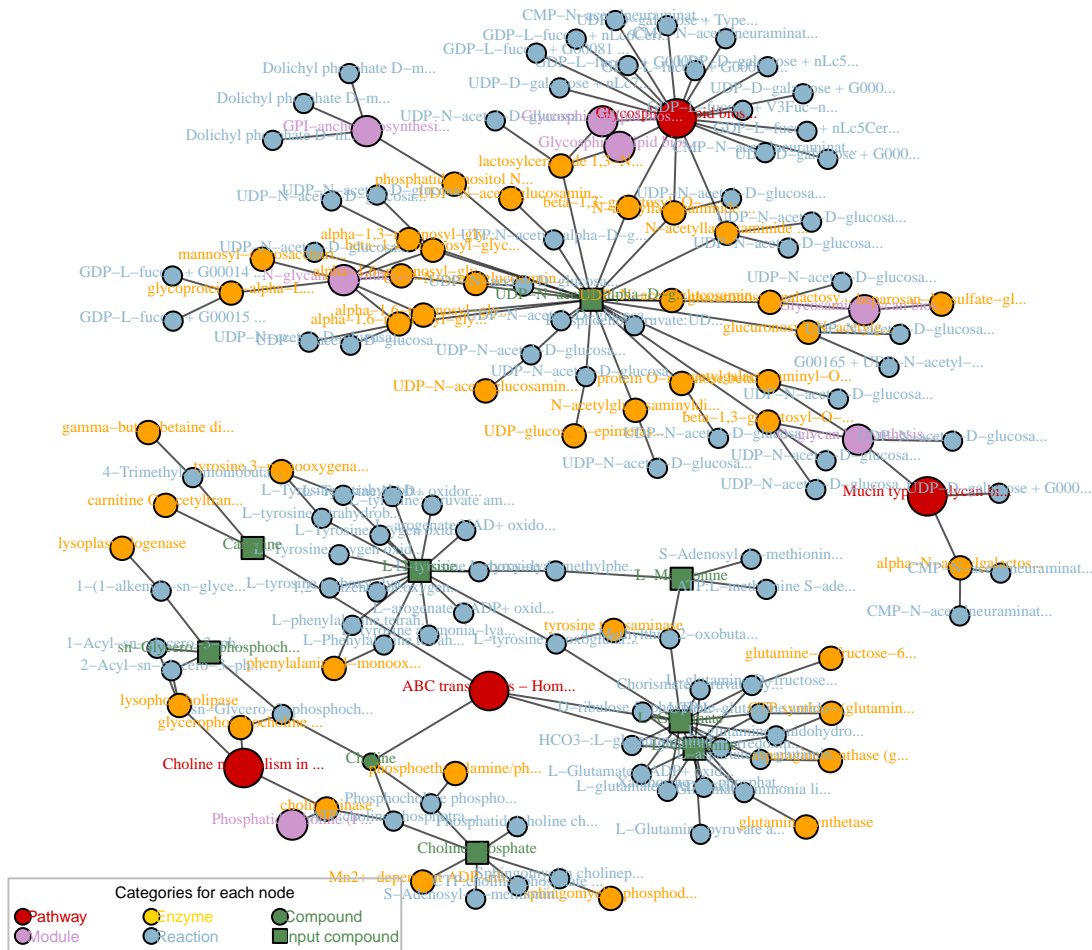

Node limit: 150

Supplement: Supplementary file 5 — Descriptive files on the three human datasets: a summary of the inputs (descriptive_input.csv), input and reported subgraph in each dataset (dataset_input.csv, dataset_subgraph.csv and dataset_subgraph.pdf), hits discussed in the results section (descriptive_hits.csv). Also contains the database object (fella_data.RData) and metadata about the database (info_fella_data.txt), the KEGG version (info_kegg.txt) and the R session (info_session.txt). (ZIP 525 kb) [file 12859_2018_2487_MOESM5_ESM.zip › fella_sm/epithelial_subgraph.pdf]
